# Characterization of diabetic peripheral neuropathy in a high-fat diet mouse model

**DOI:** 10.1371/journal.pone.0356285

**Published:** 2026-09-01

**Authors:** Guillaume Rastoldo, Alexis Chirpaz, Sarrah Tréport, Anne-Yael Gamin, Ali K. Jaafar, Aurélie Paulo-Ramos, Thomas Sandaye, Katy Thouvenot, Matthieu Bringart, Marie-Paule Gonthier, Gilles C. Lambert, Steeve Bourane

**Affiliations:** UMR 1188 Diabète athérothrombose Thérapies Réunion Océan Indien (DéTROI), Université de La Réunion, INSERM, Saint-Pierre, La Réunion; Indiana University School of Medicine, UNITED STATES OF AMERICA

## Abstract

Diabetic peripheral neuropathy (DPN) is characterized by progressive and symmetrical sensory alterations and constitutes one of the earliest and main complications of diabetes. Several mouse models have been developed to mimic the pathogenesis of DPN with some discrepancies to fully recapitulate the human disease. In the present study, we aimed to characterize the onset and progression of peripheral neuropathy in mice fed with 45% kcal High Fat Diet (HFD) for 16 weeks. Our data show that mice fed with this diet developed obesity, dyslipidemia, hyperglycemia, impaired glucose tolerance and insulin resistance. Using sensory tests, we found that HFD-fed mice developed mechanical hypoalgesia and thermal hyperalgesia starting after 12 weeks of diet. Our analysis of glabrous foot skin innervation revealed a decrease in intraepidermal nerve fiber density (IENFD) associated with a significant reduction of nociceptive Schwann cells (nSCs) in neuropathic mice after 16 weeks of HFD. Overall, this study shows that mice fed with a 45% kcal HFD recapitulate the main metabolic, behavioral and anatomical changes generally observed in patients with DPN and represent a reliable model to study the pathogenesis of the disease.

## Introduction

One of the first and main complications of diabetes is diabetic peripheral neuropathy (DPN), affecting up to 50% of diabetic patients [1,2]. DPN involves damage to the peripheral nervous system, i.e., the sensory and motor nerves as well as the autonomic nervous system that controls our organs. The most common symptom of DPN is an impairment of sensitivity to pain and touch [3]. The early stages of the pathology are characterized by abnormal sensory symptoms such as pain, tingling, burning sensations, electrical discharges, pain evoked by non-painful mechanical stimuli (allodynia), increased sensitivity to noxious stimuli (hyperalgesia) [3] significantly altering quality of life. Other sensory deficits such as numbness are also observed with progressive loss of sensation in lower limbs often leading to tissue damage with the development of ulcers, increasing the risk of amputation [4,5]. Reduced sensations in the foot also lead to progressive loss of balance resulting in an increased risk of falls [6,7].

Several mouse models have been used to study the physiopathology of DPN, from genetic and spontaneous models (WBN/Kob rats, Akita Ins2 mice, BKS-db/db mice) to alloxan- and streptozotocin (STZ) induced type 1 diabetes (T1D) or mice fed with a high-fat diet (HFD) to induce prediabetes and type 2 diabetes (T2D) (reviewed in [8,9]). Unfortunately, the diversity of mouse models used in DPN studies makes it difficult to fully understand the pathophysiological mechanisms underpinning this complication. For instance, HFD-fed mice display substantial variability in sensory deficits, impaired glucose tolerance and dyslipidemia depending on the type of diet, its composition, its duration and the mouse strain used [10,11]. There is therefore a need for standardization of HFD protocols to facilitate cross-study comparisons and determine the most suitable DPN model for preclinical studies.

So far, studies have paid much attention to the damage of small non-myelinated nerve fibers (C-fibers) responsible for sensitivity to heat and pain. The degeneration and regeneration of these fibers are typical features of diabetes and skin biopsy analyses serve as late-stage confirmations of DPN presence [12]. While C-fibers were traditionally believed to terminate freely in the epidermis, recent work by Abdo et al. challenged this concept [13]. Their findings reveal a close connection between C-fibers and specialized nociceptive Schwann cells (nSC) capable of independently generating pain signals. These cells are currently identified by the expression of S100β and Sox10 [14–16] and most importantly, eliminating nSC is sufficient to induce neuropathic pain in mice [16]. A recent report has demonstrated a decrease in nSC density in streptozotocin-induced T1D [14] but no study has evaluated the integrity of these cells in a T2D mouse model.

In the present study, we used 45% kcal High Fat Diet fed mice to recapitulate major clinical features of prediabetes and T2D [17–19]. Following the recommendations of the Diabetic Neuropathy Study Group of the European Association for the Study of Diabetes (Neurodiab), we assessed three key features of human diabetic neuropathy: nocifensive behavior, nerve conduction velocity, and intraepidermal nerve fiber density (IENFD). In addition, we investigated the integrity of nSCs, a population of glial cells recently identified as key contributors to mechanical pain. Here, we show that mice fed with a HFD develop obesity, dyslipidemia, hyperglycemia, impaired glucose tolerance as well as insulin resistance. Our behavioral analyses reveal that HFD-fed mice exhibit mechanical hypoalgesia and thermal hyperalgesia starting after 12 weeks of diet. Furthermore, our analyses uncover a decrease in intraepidermal nerve fiber density associated with a significant reduction in the number of nociceptive Schwann cells after 16 weeks of HFD. These results indicate that HFD-fed mice constitute an adequate animal model to study the development of peripheral neuropathy in T2D.

## Methods

### Animal and ethics statement

All reported experiments were performed at the GIP-CYROI platform’s animal facility, conducted in accordance with the French and European Community Guidelines for the Use of Animals in Research (86/609/EEC and 2010/63/EU), and were approved by the local Ethics Committee (n°114) for animal experimentation and the French authorities (APAFIS#31763–2018020912188806, approved on May 21, 2021). All personnel participating in this project possessed the mandatory French certification for animal experimentations and were trained in animal welfare and care. All surgeries were performed under isoflurane anesthesia and efforts were made to reduce both the number of animals used and their suffering throughout the experiment.

Twenty C57BL/6J mice (male, 6 weeks old) were purchased from Janvier (Le Genest Saint Isle, France) and housed (5 mice per cage) in a temperature-controlled environment with a 12–12h light/dark photocycle. After 2 weeks of adaptation, mice were divided in two groups (n = 10/group) and placed either on a control diet (10% kcal %fat; SF13−081) or a high-fat diet (45% kcal %fat; SF04−001) from Specialty Feeds for 16 weeks. Mice with fasting blood glucose above ≥ 200 mg/dL were considered diabetic [20].

Mice expressing a Cre recombinase under the control of the Advillin promoter [21] were crossed with Ai14 reporter mice, which carry a LoxP-flanked stop cassette excisable by Cre. In Advillin-Cre; Ai14 mice, tdTomato expression is restricted to peripheral sensory neurons (ND n = 3; HFD n = 3).

Mice expressing a tamoxifen-inducible Cre recombinase under the control of the PLP promoter (PLP-CreERT2; [22]) were crossed to Ai14 mice, which have a LoxP-flanked stop cassette excisable by Cre. At 6 weeks of age, the PLP-CreER^T2^; Ai14 progeny were injected intraperitoneally with tamoxifen (100 µg/g of mice) for 5 consecutive days. Tamoxifen was prepared in a mixture of 9/10 corn oil and 1/10 ethanol, and stored at 4 °C in a light-proof container. After 2 weeks, the mice were euthanized and the tissue were collected for immunostaining (n = 3).

### Genotyping

Genotyping of progeny from PLP-CreER^T2^ and Ai14 mice was performed by PCR using CreDN2 (GAT CTC CGG TAT TGA AAC TCC AGC), CreUP2 (GCT AAA CAT GCT TCA TCG TCG G), Ai14mut-Fwd (CTG TTC CTG TAC GGC ATG G) and Ai14mut-Rev (GGC ATT AAA GCA GCG TAT CC) primers.

### Oral glucose tolerance test (OGTT)

An OGTT was performed after 16 weeks of diet in all mice. After 6 hours of fasting, one drop of tail blood was collected and glycemia was analyzed using a standard glucometer (One Touch Profile, Lifescan Inc., Milpitas, CA, USA). Oral gavage with glucose 30% (2 g/kg body weight) into conscious mice was performed and glycemic levels measured 15, 30, 45, 60, 90 and 120-min post gavage.

### Nerve conduction velocity

After 16 weeks of diet, mice were anesthetized with isoflurane (IsoFlo^®^, Centravet, France) and placed on a heating pad. Once anesthetized, motor nerve conduction velocities (MNCV) and sensory nerve conduction velocities (SNCV) were determined as previously described [23–26]. Using the Keypoint 4, Dantec-Medtronic electroneuromyography device, we performed both proximal and distal nerve stimulations of the sciatic nerve, registering the transmitted potentials with sensing electrodes at the gastrocnemius muscle. We used stainless steel subdermal needle electrodes to deliver single square-wave supramaximal stimulation with 0.2 millisecond impulses. MNCV was calculated by subtracting the distal from the proximal latency (measured in milliseconds) from the stimulus artifact of the take-off of the evoked potential, and the difference was divided into the distance between both stimulating electrodes (measured in millimeters using a Vernier caliper). For SNCV, recording electrodes were placed on the dorsum of the foot and stimulating electrodes on the ankle. The latency of onset (milliseconds) of the sensory nerve action potential after supramaximal antidromic stimulation of the sural nerve at the ankle was divided by the distance between the recording and stimulation electrodes.

### Tissue Harvest

After 12 or 16 weeks of diet, mice received a subcutaneous injection of buprenorphine (0.05 mg/kg, Buprecare^®^, Centravet, France) and were anesthetized with isoflurane (3%). Mice were then euthanized by intracardiac puncture and blood samples were collected and centrifuged at 2000 g for 10 min at 4°C. Serum was stored at −80°C until use. Intracardiac perfusion was performed with 20 mL of phosphate buffered saline (PBS) and 20 mL of 4% paraformaldehyde (PFA) dissolved in PBS. The hind-paw plantar surfaces and DRG of each mouse were collected and post-fixed overnight in 4% PFA solution. Biopsies of foot skin and dorsal root ganglia (DRG) were then rinsed and cryoprotected into 30% sucrose dissolved in PBS at 4°C. The samples were then embedded in optimal cutting temperature (OCT, Tissue-Tek®, USA) compound and stored at −80°C until sectioning.

### Immunohistochemistry

Hind-paw plantar surface (30 μm) and DRG (14 μm) sections were collected onto glass slides and dried on a heating plate for 10 minutes. The slides were stored at –20°C until use. Foot skin sections were rehydrated (3x10min) in PBS. Blocking and permeabilization were done by incubation (1h) in superblock (Thermo Fisher) with 1.5% donkey anti-serum and 0.5% Triton X-100. For intraepidermal nerve fibers density, sections were incubated with the primary antibody anti-PGP9.5 (1:500, #14730–1-AP, ProteinTech) overnight at 4°C and the next day at room temperature. To identify nociceptive Schwann cells, the same protocol was applied with L1 cell adhesion molecule (L1CAM, 1:500, (#MAB5272, R&D System), Sox10 (1:200, #AF2864 R&D systems), S100β (1:500, #S2532, Sigma-Aldrich) and Ds-red (1:1000, #632496, Takara bio) antibodies but the sections were incubated only once overnight at 4°C. Sections were then rinsed in PBT (PBS with 0.1% triton) and incubated for 1 h at room temperature with secondary antibody and DAPI (0.5 μg/mL) before being mounted and kept at 4 °C. For DRG, the same protocol was used with primary antibodies against CGRP (1:1000, #ab36001, Abcam), IB4 (1:300, #I32450, ThermoFisher) and TRPV1 (1:1000, #Acc-030, Alomone). The following secondary antibodies were used: Alexa Fluor 594 donkey anti-rabbit (1:500, Abcam; #ab150064), Alexa Fluor 488 donkey anti-mouse (1:500, Invitrogen; #A-21202), Alexa Fluor 488 donkey anti-rat (1:500, Invitrogen; # A-21208), Alexa Fluor 647 donkey anti-goat (1:500, Invitrogen, #A-31573). A complete list of antibodies is provided in Table 1.

**Table 1:** Primary, secondary antibodies, and 4′,6-diamidino-2-phenylindole (DAPI).

Antigen	Host	Dilution	Supplier
PGP9.5 (#14730–1-AP)	Rabbit	1:500	ProteinTech
L1CAM (#MAB5272)	Rat	1:500	R&D System
Sox10 (#AF2864)	Goat	1:200	R&D systems
S100β (#15145–1-AP)	Rabbit	1:500	Proteintech
Isolectin GS-IB4 (#I32450)	/	1:300	ThermoFisher
CGRP (#ab36001)	Goat	1:1000	Abcam
TRPV1 (#Acc-030)	Rabbit	1:1000	Alomone
Ds-red (#632496)	Rabbit	1:1000	Takara bio
Alexa Fluor 594 (#ab150064)	Donkey anti-rabbit	1:500	Abcam
Alexa Fluor 647 (#A-31573)	Donkey anti-goat	1:500	Invitrogen
Alexa Fluor 488 (#A-21208)	Donkey anti-rat	1:500	Invitrogen
DAPI (#10236276001)	/	1:1000	Sigma

### Identification and quantification of nociceptive Schwann cells

Nociceptive Schwann cells were identified according to Hu et al. criteria [14]. Commonly, nociceptive Schwann cells connect to PGP9.5+ nerve endings and are located within 25 µm depth in the subepidermal zone at the junction between dermis and epidermis. For quantification, we excluded L1CAM positive staining in glands or large peripheral fiber bundles found deeper in the dermis. The number of nSCs was reported per mm length of foot skin. To quantify nSCs cellular extensions, we measured cell fluorescence using ImageJ software. Mean fluorescence intensity was measured at the subepidermal zone in each image of the foot skin within the region of interest.

### Confocal imaging

Intraepidermal nerve fibers density and nociceptive Schwann cells number were evaluated under a laser scanning confocal microscope Eclipse (Nikon) with a 40x objective. Following immunostaining, 30 µm thick Z-stack images were taken and analyzed on orthogonal maximum intensity projection of the original confocal Z-stack images.

### Metabolic markers

Plasma hemoglobin A1C (HbA1c) levels were determined using DCA® Vantage Analyzer (Siemens Healthineers, France) and the mouse HbA1c reagent kit (#23−312018). Serum triglycerides (TG) and total cholesterol (TC) levels were determined using colorimetric assays (Diasys®, #157109910021 and #113009910021, Germany). Plasma insulin and leptin concentrations were measured using ELISA kits (Mercodia®, #10-1247-01, Sweden and RayBiotech®, Peachtree Corners, GA, USA). All metabolic markers were measured after 16 weeks of diet.

### Behavioral tests

#### Von Frey test.

Mice were familiarized with the testing apparatus 1h/day during a week before behavioral testing. Animals were placed on an elevated wire grid and the plantar surface of the hindpaw was stimulated with calibrated von Frey monofilaments (0.008–6 g). The paw withdrawal threshold for the von Frey assay was determined by SUDO up-down method [27]. Testing was performed every 4 weeks after the start of the diet and up to 16 weeks.

#### Hargreaves test.

To measure radiant heat pain by Hargreaves test [28], animals were put in plastic boxes and the plantar paw surface was exposed to a beam of radiant heat according to the Hargreaves method. Paw withdrawal latency was then recorded and beam intensity was adjusted to result in a latency around 10 seconds in control animals. Testing was only initiated after exploratory and grooming behaviors had ended. High speed of skin heating (6.5°C/sec) preferentially activates Aδ-fibers [29]. Based on previous study [30], we used a medium to high speed of temperature heating (5°C/sec during 10 sec to obtain 50°C) to predominantly activate Aδ-fibers. Heat stimulation was repeated 5 times with an interval of 10 min for each animal and the mean was calculated. A cutoff time of 30 seconds was set to prevent tissue damage. Testing was performed every 4 weeks after the start of the diet and up to 16 weeks.

#### Brush test.

To measure dynamic allodynia, mice were familiarized with the testing apparatus (the same that for von Frey test) 1h/day during a week before testing. A soft paintbrush (Lefranc & Bourgeois® - Brush no. 4 #175160) was used to gently stroke the plantar surface of the hindpaw in the direction from heel to toe. The test was repeated five times, with intervals of 5 min. For each test, no evoked movement was scored as 0, and walking away or occasionally brief paw lifting (~1 sec or less) was scored as 1. For each mouse, the cumulative scores of the five tests were used to form a percentage response (100% response equivalent to 5 evoked movements during the 5 stimulations of the paw). Testing was performed every 4 weeks after the start of the diet for up to 16 weeks.

#### Pinprick test.

To measure acute mechanical pain, mice were familiarized with the testing apparatus (the same that for von Frey test) 1h/day during a week before testing. After this acclimation, for pinprick test, we touched the plantar surface of the hindpaw with a pin [31] and measured numbers of withdrawal response as for the brush tests. For each mouse, the cumulative scores of five tests were used to form a percentage response (100% response equivalent to 5 evoked movements during the 5 stimulations of the paw). Testing was performed every 4 weeks after the start of the diet and up to 16 weeks.

### Statistical analysis

We first analyzed the normality of data and showed that the results of serum TG and TC levels, insulinemia, motor NCV, sensory NCV, IENFD and L1CAM-positive cells were normally distributed. Thus, unpaired Student’s 𝑡-tests (two-tailed) were used for statistical comparisons. For behavioral data, a two-way ANOVA for repeated measures followed by Bonferroni’s post-hoc test was used to determine statistically significant differences. A *P* value of less 0.05 was considered significant.

## Results

### Body weight gain, serum triglycerides and total cholesterol levels

**Fig 1:**
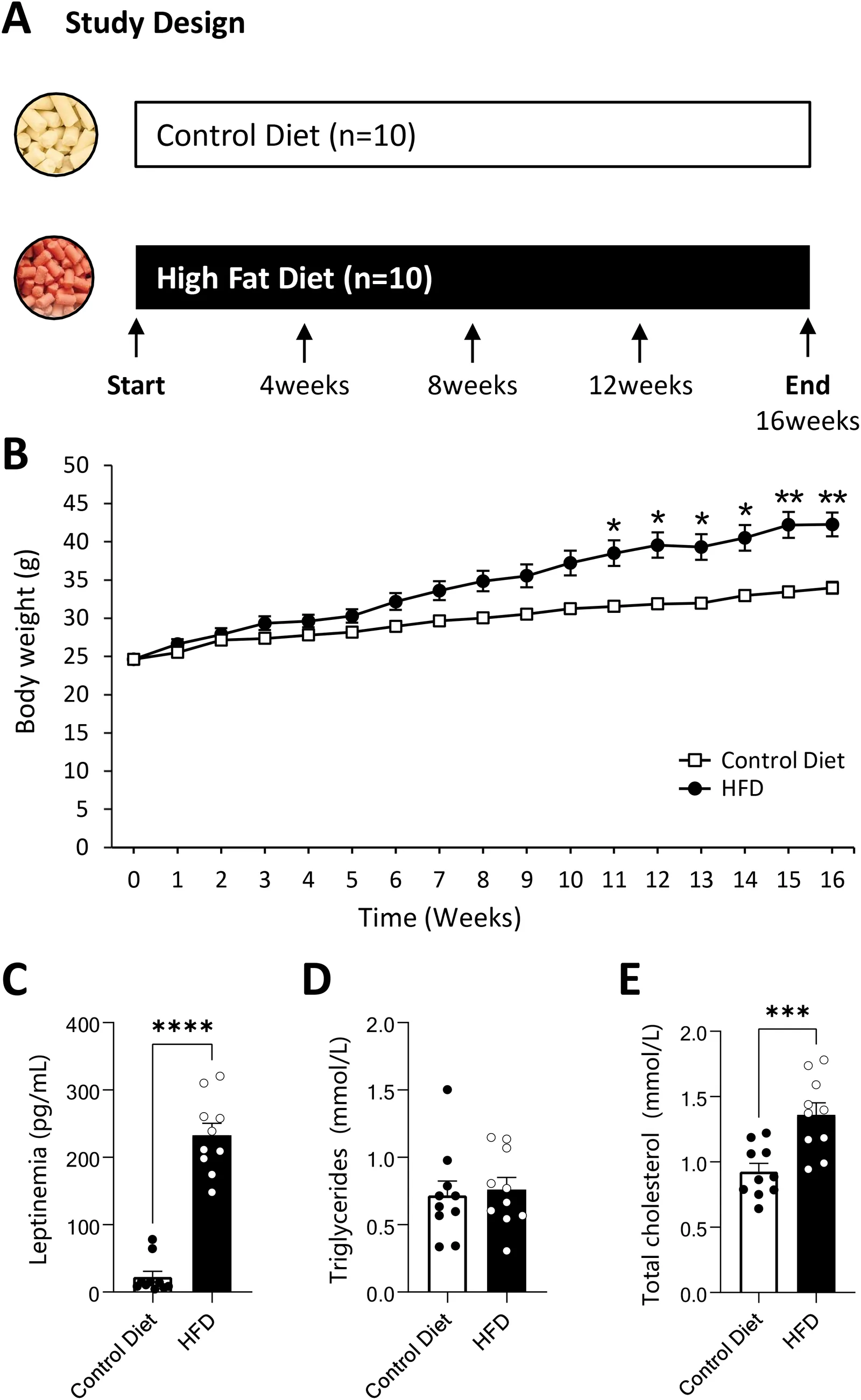
Study design and HFD-induced dyslipidemia. (A) Study Design used to assess the effect of a HFD (45% kcal % fat) vs. a control diet (10% kcal % fat) in two groups of mice (n = 10/group). (B) Body weight of control diet-fed mice and HFD-fed mice during the 16 weeks of diet. (C) Leptinemia of control and HFD mice at 16 weeks of diet. (D) Plasma triglycerides levels of control and HFD mice at 16 weeks of diet. (E) Total cholesterolemia of control and HFD mice at 16 weeks of diet. *𝑃 < 0.05, **𝑃 < 0.01, ***𝑃 < 0.001: control diet vs. HFD. Data are presented as means ± SEM.

To generate a mouse model of T2D, we fed C57Bl/6J male mice with a control diet (10% kcal from fat) or a HFD (45% kcal from fat) for 16 weeks (n = 10/group, Fig 1A). As anticipated, HFD-fed mice gained significantly more weight than controls. The difference became significant from week 11 onward (Fig 1B). In line with weight gain, blood samples analyses revealed that HFD-fed mice exhibited higher circulating levels of leptin compared with controls (Fig 1C). TG levels were not significantly increased (Fig 1D). However, HFD-fed mice showed significantly higher plasma TC levels than mice fed the control diet (𝑃 < 0.01; Fig 1E).

### Glucose homeostasis/ Glucose regulation

**Fig 2:**
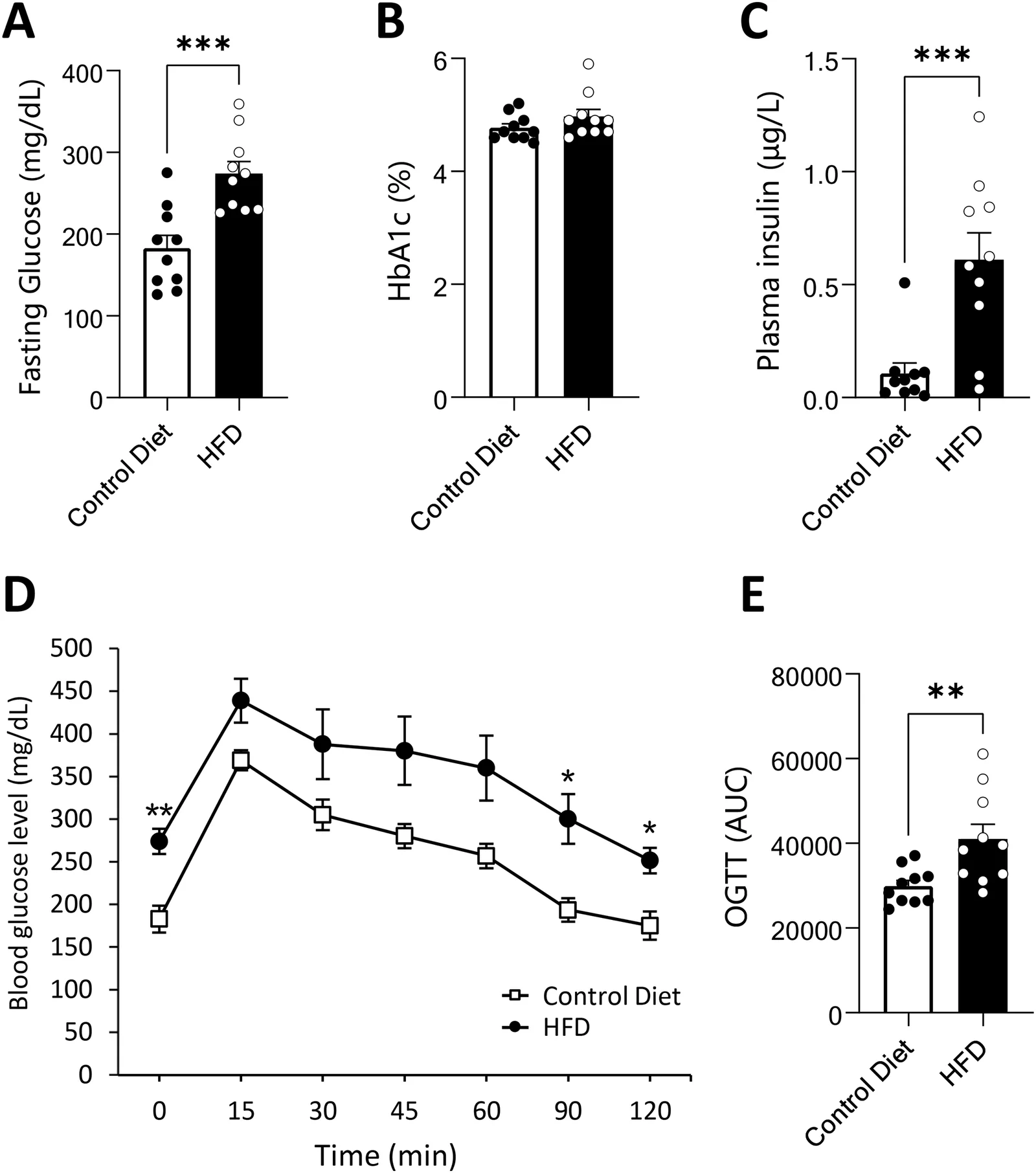
HFD-induced impaired glucose tolerance. (A) Fasting blood glucose of control diet-fed mice and HFD-fed mice at 16 weeks of diet. (B) Percentage of glycated hemoglobin (HbA1c) of control diet-fed mice and HFD-fed mice at 16 weeks of diet. (C) Plasma insulin of control diet-fed mice and HFD-fed mice at 16 weeks of diet. (D) Oral glucose tolerance test (OGTT) curves performed at 16 weeks of diet in control and HFD-fed mice. (E) Area under the curve (AUC) during OGTT for control diet-fed mice and HFD-fed mice. *𝑃 < 0.05, **𝑃 < 0.01, ***𝑃 < 0.001: control diet vs. HFD. Data are presented as means ± SEM (n = 10/group).

Fasting glucose levels exhibited a significant increase in HFD-fed mice compared with controls (𝑃 < 0.001; Fig 2A) whereas HbA1c levels were not significantly altered (Fig 2B). In contrast, plasma insulin levels in HFD-fed mice were significantly increased compared with controls (𝑃 < 0.001; Fig 2C) indicating impaired glucose tolerance and insulin resistance. To confirm this observation, we performed an oral glucose tolerance test (OGTT) after 16 weeks of diet on both groups and observed that glucose tolerance was indeed impaired (Fig 2D) with the AUC of blood glucose concentrations significantly higher in HFD-fed mice compared with controls (𝑃 < 0.01; Fig 2E).

### Sensory tests and NCV analysis

**Fig 3:**
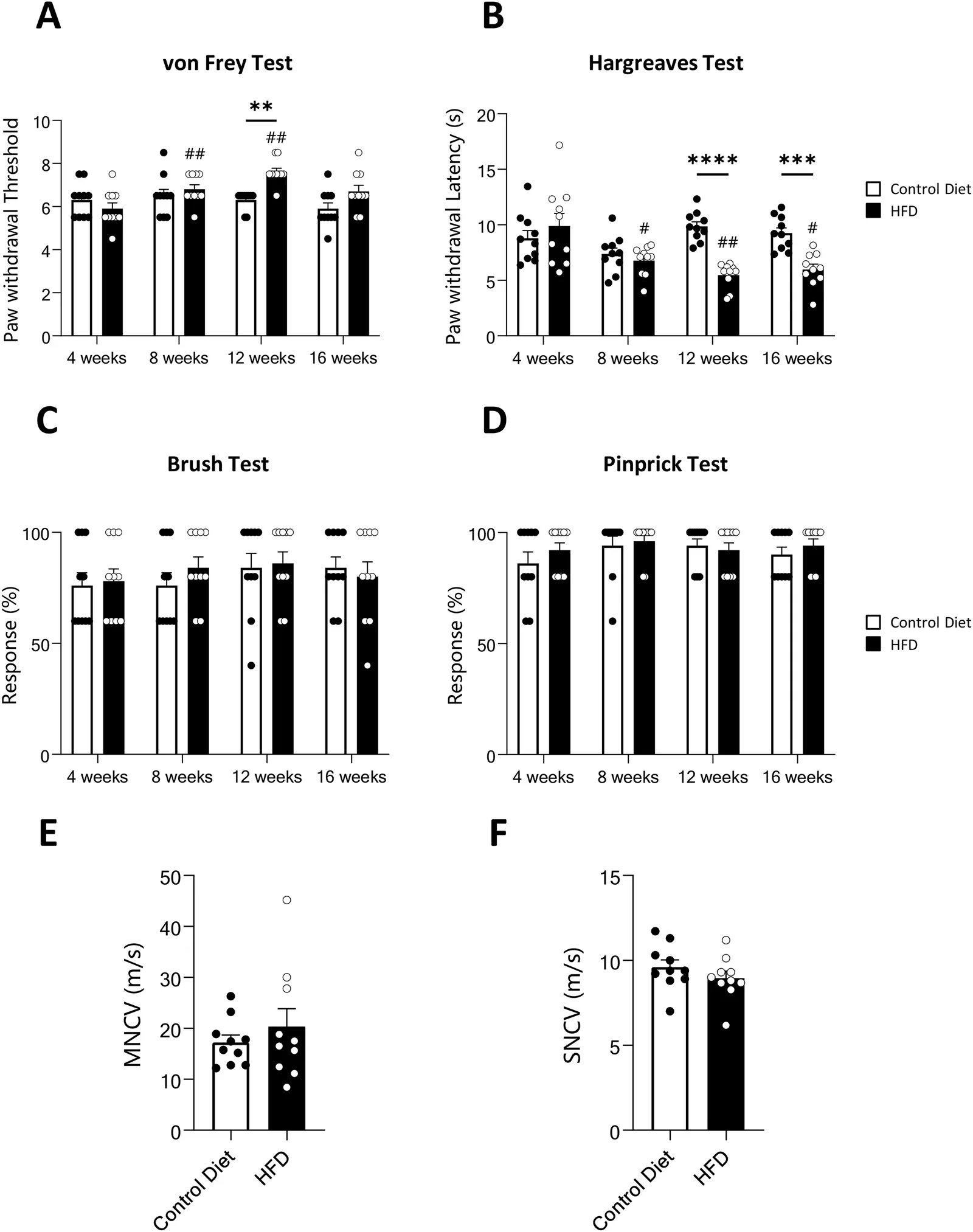
HFD-induced sensory deficits related to diabetic neuropathy. (A) Quantification of static mechanical sensitivity was assessed every 4 weeks in control and HFD-fed mice using von Frey filaments. (B) Quantification of thermal sensitivity by Hargreaves test every 4 weeks in control and HFD-fed mice. (C) Quantification of haptic mechanical sensitivity by brush test every 4 weeks in control and HFD-fed mice. (D) Quantification of acute mechanical pain by pinprick test every 4 weeks in control- and HFD-fed mice. (E) Motor nerve conduction velocity (MNCV) performed at 16 weeks of diet in control and HFD-fed mice. (F) Sensory nerve conduction velocity (SNCV) performed at 16 weeks of diet in control and HFD-fed mice. **𝑃 < 0.01, ***𝑃 < 0.001: control diet vs. HFD. #𝑃 < 0.05, ##𝑃 < 0.01: HFD 4 weeks vs. HFD 8, 12 or 16 weeks. Data are presented as means ±SEM (n = 10/group).

To evaluate peripheral neuropathy, we performed various behavioral sensory tests every 4 weeks for 16 weeks. HFD-fed mice exhibited an increase in paw withdrawal threshold with the von Frey test as early as 8 weeks (4 weeks HFD vs. 8 weeks HFD, 𝑃 < 0.01, Fig 3A) and were significantly different from their control diet-fed counterparts after 12 weeks of diet (12 weeks control diet vs. 12 weeks HFD, 𝑃 < 0.01, Fig 3A), indicating a decrease in mechanical sensitivity. In parallel, we observed an increase in thermal sensitivity of HFD-fed mice with the Hargreaves test compared with control mice from week 8 (Fig 3B). The latency of hind-paw withdrawal in response to noxious thermal stimulus was significantly decreased in HFD-fed mice compared with control diet-fed mice at 12 and 16 weeks (𝑃 < 0.001). No significant change in haptic mechanical sensitivity (Fig 3C) evaluated with the brush test or acute mechanical pain sensitivity (Fig 3D) assessed by the pinprick test was noticed over 16 weeks between both groups. In order to evaluate the integrity of peripheral nerves in control and HFD mice, we measured nerve conduction velocity (NCV) of the sciatic and sural nerves of each animal at 16 weeks of HFD. Our results did not show any significant difference in both motor (Fig 3E) and sensory (Fig 3F) NCV in HFD-fed mice compared with controls.

### Intraepidermal innervation

**Fig 4:**
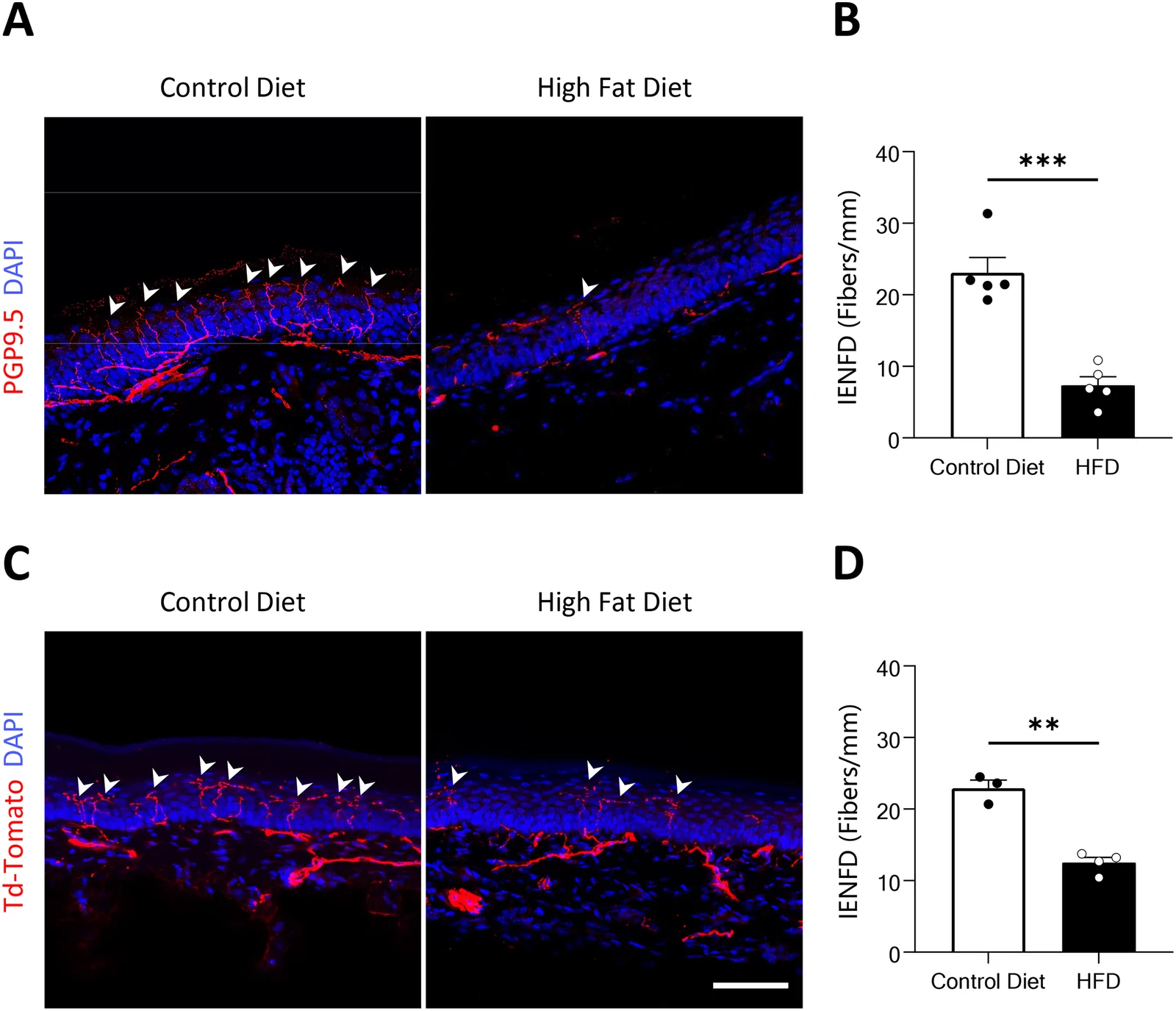
Nerve fiber degeneration in the foot skin of 16 weeks HFD-fed mice. (A) PGP9.5 immunofluorescence and DAPI staining in foot skin sections from control and HFD-fed mice. Arrowheads indicate nerve fibers in the epidermis of the foot skin. (B) Quantification of IENFD in foot skin section of control (n = 5) and HFD-fed mice (n = 5) presented as the number of fibers/mm of epidermis. (C) Tdtomato immunofluorescence with DAPI staining in foot skin section of Advillin-Cre;Ai14 double transgenic mouse under control diet and HFD. (D) Quantification of IENFD in Advillin-Cre; Ai14 foot skin section of control (n = 3) and HFD-fed mice (n = 4). **𝑃 < 0.01, ***𝑃 < 0.001: control diet vs. HFD. Data are presented as means per mouse ± SEM. Scale bar: 50 μm.

To fully characterize the neuropathic phenotype in our model, we analyzed the intraepidermal nerve fiber density in the glabrous skin of control and HFD-fed mice using immunostaining against PGP9.5. We did not observe any significant difference in IENFD after 12 weeks of HFD (S1A Fig). However, we found a significant reduction of foot skin innervation in HFD-fed mice compared with control mice after 16 weeks of diet (𝑃 < 0.001; Fig 4A, 4B). To confirm this observation, we used a genetic approach to label sensory terminal endings in the skin using Advillin-Cre; Ai14 mice that express tdTomato in peripheral sensory neurons. The results confirmed our previous observation with a significant reduction of tdTomato positive sensory fibers in the epidermis of HFD-fed mice compared to control fed-mice after 16 weeks of diet (P < 0.01; Fig 4C, 4D).

### Nociceptive Schwann cells

**Fig 5:**
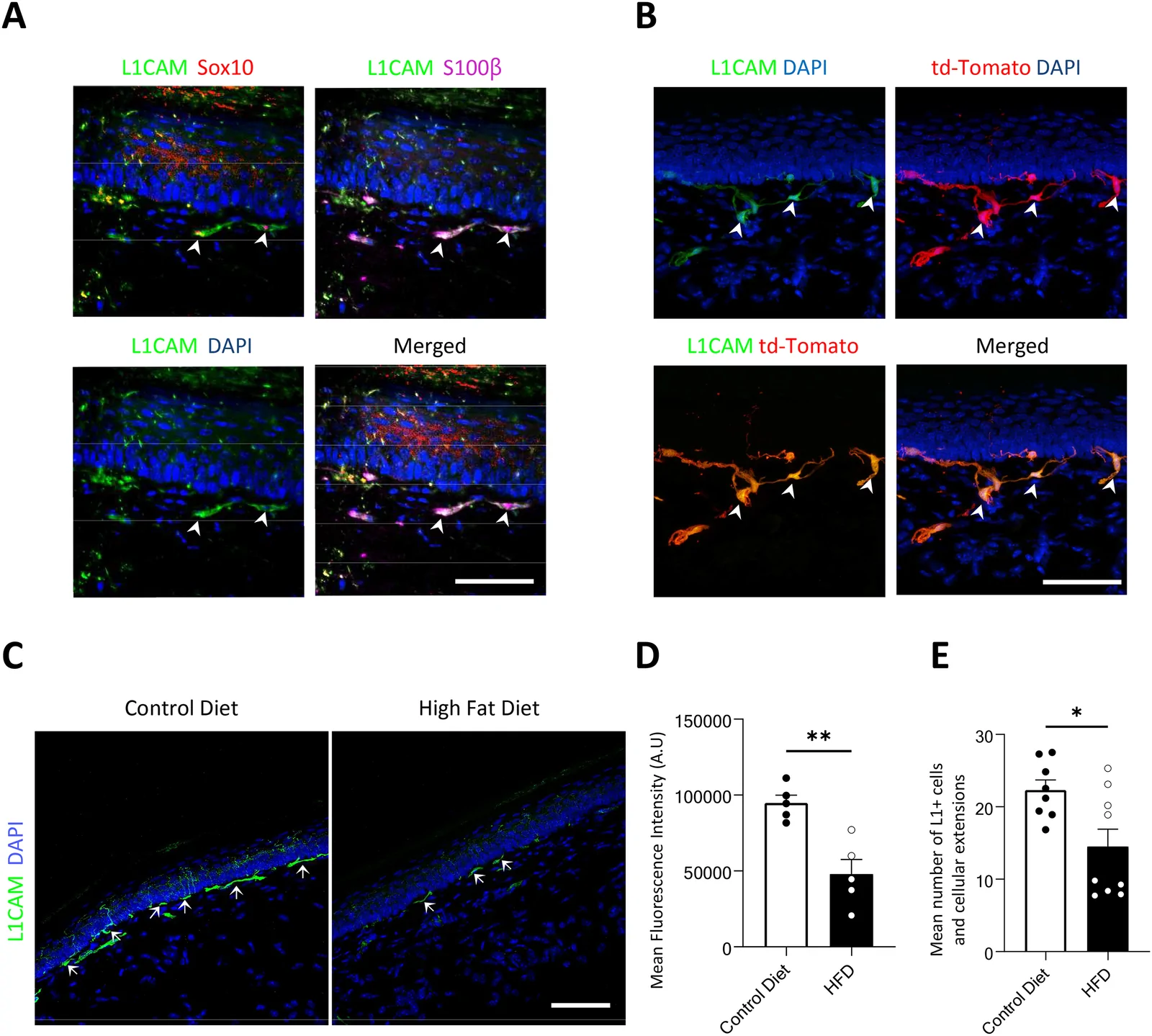
Nociceptive Schwann cells degeneration in the foot skin of 16 weeks HFD-fed mice. (A) L1CAM immunofluorescence colocalized with Sox10 and S100β classical markers of nociceptive Schwann cells in the foot skin. (B) L1CAM immunofluorescence colocalized with tdTomato expressing nociceptive Schwann cells in a tamoxifen-induced PLP-CreERT2;Ai14 double transgenic mouse. Arrowheads indicate nociceptive Schwann cells and their cellular extensions at the border of the epidermis and dermis. (C) L1CAM immunofluorescence with DAPI staining in foot skin section of control and HFD-fed mice. Arrows indicate nociceptive Schwann cells and their cellular extensions at the border of the epidermis and the dermis. (D) Mean fluorescence intensity quantification of L1CAM immunostaining at the localization of nociceptive Schwann cells (at the border of epidermis and dermis) in control diet (n = 5) and HFD mice (n = 5). (E) Quantification of L1CAM-positive cells and their cellular extensions presented in number/mm of epidermis in control diet (n = 8) and HFD mice (n = 9). *𝑃 < 0.05, **𝑃 < 0.01: control diet vs. HFD. Data are presented as means per mouse ± SEM. Scale bar: 50 μm.

Nociceptive Schwann cells (nSCs) have recently been identified to play a critical role as support for terminal nerve fibers and to contribute to mechanical and neuropathic pain sensation [13,16]. Given that double-immunostaining for S100β and Sox10 markers, a method conventionally used to identify this specific Schwann cells subpopulation, did not allow optimal visualization of nSCs in the foot skin of mice, we looked for other markers. By screening for the expression of known markers of Schwann cells subtypes, we observed that L1CAM a marker of non-myelinating SCs [32], was highly expressed in nSCs (Fig 5A). We showed that L1CAM colocalizes with both Sox10 and S100β markers [14–16] (Fig 5A), and verified the expression of L1CAM in nSCs by using PLP-CreERT2;Ai14 double transgenic mice that express tdTomato specifically in this cell type (Fig 5B) [22,33]. Thus, immunostaining against L1CAM is a good alternative to the double immunostaining against Sox10 and S100β to identify nSCs in the foot skin. Using the L1CAM immunostaining approach, we did not find any significant change in the number of nSCs after 12 weeks of HFD (S1B Fig). In contrast, both the mean fluorescence intensity (Fig 5C,D) and the mean number of L1CAM-positive nSCs (Fig 5C,E) were significantly decreased in 16 weeks HFD-fed mice compared to controls (𝑃 < 0.001 and 𝑃 < 0.01 respectively). These observations indicate a decrease in number of both sensory nerve endings and nociceptive Schwann cells in the foot skin of 16 weeks HFD-fed mice.

### Nociceptive neurons subtypes

**Fig 6:**
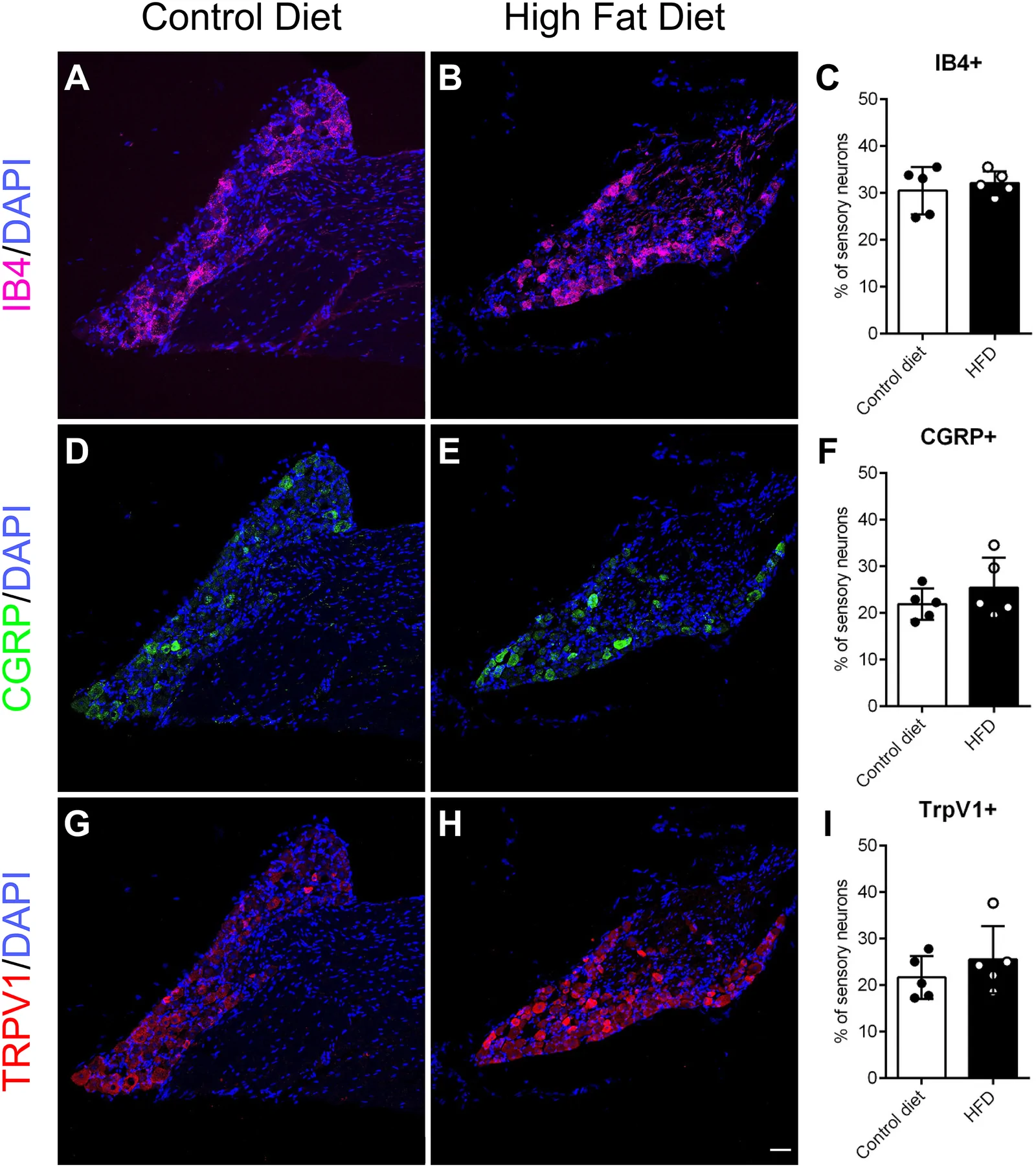
Analysis of nociceptive neurons subtypes in the DRG of 16 weeks HFD-fed mice. (A-B) IB4 and DAPI staining in DRG section of control and HFD-fed mice. (C) Quantification of percentage of IB4-positive neurons in the DRG of control (n = 5) and HFD-fed mice (n = 5). (D-E) CGRP and DAPI staining in DRG section of control and HFD-fed mice. (F) Quantification of percentage of CGRP-positive neurons in the DRG of control (n = 5) and HFD-fed mice (n = 5). (G-H) TRPV1 and DAPI staining in DRG section of control and HFD-fed mice. (I) Quantification of percentage of TRPV1-positive neurons in the DRG of control (n = 5) and HFD-fed mice (n = 5). Data are presented as means per mouse ± SEM. Scale bar: 50 μm.

In order to analyze if HFD has an impact on sensory neurons involved in pain sensation, we quantified the number of nociceptive neurons subtypes in the dorsal root ganglia of 12 and 16 weeks HFD-fed mice. We used IB4 and CGRP staining to identify non-peptidergic and peptidergic nociceptive sensory neurons, respectively. We did not observe any significant change in the number of non-peptidergic (Fig 6 A-C, S1C Fig,) and peptidergic (Fig 6 D-F, S1D Fig) nociceptive neurons after 12 and 16 weeks of HFD. Given that the most robust and persistent behavioral phenotype observed in our model was thermal hyperalgesia, we analyzed the expression of TRPV1, an ion channel known to be involved in thermosensation [34]. We observed a significant increase in the number of neurons expressing TRPV1, at 12 weeks (S1E Fig) but not at 16 weeks post HFD (Fig 6 G-I). Altogether, these observations indicate that nociceptive neurons subtypes in the DRG are preserved after 12 and 16 weeks of HFD with a transient increase in TRPV1 expression only after 12 weeks of diet.

## Discussion

In the present study, we used a 45% kcal HFD-fed mice for 16 weeks to generate a mouse model of diabetic peripheral neuropathy. We observed that HFD-fed mice display increased weight gain, elevated leptinemia, hypercholesterolemia, impaired glucose tolerance as well as elevated fasting blood glucose and insulin resistance, recapitulating most of the major metabolic dysfunctions observed in T2D. Analysis of IENFD in the foot skin of these animals revealed an important reduction in both terminal nerve fibers and nSCs in 16 weeks HFD-fed mice. These neuroglial changes coincided with clear sensory dysfunctions, as evidenced by impaired responses to light mechanical stimuli and exaggerated thermal sensitivity, consistent with a peripheral neuropathic phenotype.

The HFD-fed mice model used in the present study recapitulates most of the metabolic traits of T2D, except for elevated TG and HbA1c, two parameters that usually appear when the animals are maintained for more than 16 weeks on HFD [35,36]. To characterize the progression of peripheral neuropathy, we assessed three key features present in human pathology (nocifensive behavior, nerve conduction velocity, and IENFD) considering that the presence of alterations in two of these parameters is sufficient to establish a neuropathic phenotype in rodents [37]. We demonstrate that HFD-fed mice exhibit a unique behavioral phenotype with mechanical hypoalgesia and thermal hyperalgesia coupled with a severe loss of intraepidermal nerve fibers but no impairment of motor or sensory nerve conduction velocity. The absence of significant NCV slowing in our study is consistent with several reports showing that electrophysiological deficits may emerge only at later stages of DPN [38,39]. This dissociation between functional sensory changes and large-fiber electrophysiological measures further supports the idea that NCV may be relatively preserved during early stages of HFD-induced neuropathy [40]. In line with Neurodiab criteria, we can conclude that our HFD-fed mice model developed diabetic peripheral neuropathy.

We observed that mice fed with the Specialty Feeds 45% HFD display a unique phenotype with two opposite behavioral impairments: thermal hypersensitivity (hyperalgesia) and mechanical hyposensitivity (hypoalgesia). Thermal nociception is thought to be mediated by C- and Aδ-fibers in which the TRPV1 ion channel plays a critical role for the detection of noxious heat stimuli [41,42]. Here, we observed a significantly increased expression of TRPV1 in sensory neurons after 12 weeks of HFD that underpins the thermal hypersensitivity phenotype observed in our behavioral analysis and previous studies [43]. The observation that thermal hyperalgesia persists in 16 weeks HFD-fed mice even though the upregulation of TRPV1 is not maintained in addition to the loss of terminal nerve fibers in the skin indicates that other mechanisms such as central sensitization and/or the reduction of nSCs may be at play in this process [16,44].

In addition, we observed a transient decrease in responses to mechanical stimulation induced by von Frey filament indentations at 12 weeks but not 16 weeks post HFD. Von Frey filaments activate low-threshold mechanoreceptor (Aβ-LTMRs and Aδ-LTMRs) as well as nociceptive C-fibers [45]. Specifically, brief vertical von Frey stimulation activates Aβ mechanoreceptors, which are also activated by brush stimulation, while a sustained von Frey stimulation (10 seconds) is presumed to activate epidermal C- and Aδ nociceptors [46]. In our analysis, we did not observe any significant decrease in IENFD or nSCs number in the foot skin that could support the decrease in mechanical sensitivity in mice fed with HFD for 12 weeks. It is noteworthy that transient mechanical alterations have also been observed in different mouse models of diabetic neuropathy [47–49] but the cellular and molecular basis of these changes are still elusive. Future studies targeting mechanosensory neurons are necessary to understand the mechanisms underlying the variations of mechanical sensitivity during diabetes onset.

For thermal detection, electrophysiological experiments demonstrate that slow speed of skin heating (0.9°C/sec) preferentially activated C-fibers, whereas high speed of skin heating (6.5°C/sec) preferentially activated Aδ-fibers [29]. The dissociation between mechanical hypoalgesia and thermal hyperalgesia in our model likely reflects selective impairments of distinct sensory fiber populations. On HFD, rodents have been shown to develop variable phenotypes of thermal hypoalgesia [35,50–52], hyperalgesia [53–56] or mechanical allodynia [54,55,57–59], consistent with the notion that HFD-induced neuropathy reflects sensory phenotypes of prediabetes. Furthermore, the diversity of HFD composition, particularly in fat content and fat source (e.g., lard-, plant-, or fish oil-based), often leads to divergent phenotypic outcomes [17]. Although no previous work has investigated diabetic peripheral neuropathy with the exact diet we used (Specialty Feeds 45% fat), this diet is equivalent to the widely studied Research Diets D12451 [35,36,50,53,54,56–58]. However, despite this equivalence, we observed substantial differences in sensory deficits, suggesting that even small variations in diet composition could significantly influence experimental results [60].

To identify possible damages to small sensory fibers, we also performed a quantification of intraepidermal nerve fibers density [61]. Damage to small sensory fibers is one of the earliest manifestations of DPN that can be observed in prediabetic conditions [62]. We found a significant reduction in epidermal innervation in the hind paw of 16 weeks HFD-fed mice versus controls, in line with previous reports [35,50,58]. Of note, reduction in the number of intraepidermal nerves fibers was observed using two different approaches: (i) PGP9.5 immunostaining and (ii) advillin-tdTomato sensory terminal fibers labeling, confirming the specificity of HFD-induced axonal degeneration of peripheral sensory neurons at 16 weeks. It has been established that small nerve fibers penetrating the epidermis are mostly nociceptive [12] and that these fibers undergo degeneration and regeneration at the early stages of type 2 diabetes in humans [6,63]. Until recently, it was thought that because they lack the protection and nutrient supply, that myelinated Schwann cells provide to A-fibers, unmyelinated C-fibers were more susceptible to damage at the early stages of diabetic neuropathy. However, a specialized cutaneous type of Schwann cells, termed nociceptive Schwann cells, in close relationship with nociceptive C-fibers in the dermis has recently been discovered [13]. These cells contribute to terminal axon support and play critical roles in skin mechano-sensitivity and neuropathic pain [15,16,64,65]. Therefore, nociceptive endings in the epidermis are not free but form a mesh-like organ with a mutual dependence between C-fibers and nSCs. The absence of nSCs induces a retraction of epidermal nerve fibers and development of mechanical, cold, and heat hyperalgesia [16]. In the present study, we identified L1CAM as a reliable marker for nSCs and a good alternative to the conventional S100β/Sox10 double immunostaining method [14–16]. L1CAM immunofluorescence colocalized with tdTomato expressing nociceptive Schwann cells in a tamoxifen-induced PLP-CreERT2;Ai14 double transgenic mouse. As outlined by Hu et al., the precise localization of Schwann cells serves as a crucial criteria in distinguishing nSCs [14]. Our findings provide the first evidence of a significant reduction of nSCs and their cellular processes in early T2D, consistent with observations in mice with T1D [14].

## Conclusion

In this study, we used 45% kcal HFD to generate a mouse model of diabetic peripheral neuropathy. We observed key metabolic dysfunctions indicative of early T2D in these animals. Behavioral analysis uncovered early-onset peripheral neuropathy with mechanical hypoalgesia and thermal hyperalgesia. Interestingly, we observed a significant decrease in number of nociceptive Schwann cells and their extensions in the foot skin of neuropathic mice paralleling the reduction of terminal nerve fibers which highlights the vulnerability of these cells to T2D onset. A limitation of this study is that all experiments were performed in male mice, and potential sex-dependent differences in the development of HFD-induced peripheral neuropathy were not addressed. Nethertheless, our analyses recapitulate the early events of DPN pathophysiology, paving the way for future investigations into the molecular mechanisms underlying sensory neurons and nociceptive Schwann cells alterations and potential strategies to thwart this debilitating complication of diabetes.

## Supporting information

S1 Fig Analysis of IENFD, nSCs in the foot skin and nociceptive neurons subtypes in the DRG of 12 weeks HFD-fed mice. (A) Quantification of IENFD in foot skin section of control (n = 4) and HFD-fed mice (n = 4) presented as the number of fibers/mm of epidermis. (B) Quantification of L1CAM-positive cells presented in number of cells/mm of epidermis in control diet (n = 4) and HFD mice (n = 4). (C) Quantification of percentage of IB4-positive neurons in the DRG of control (n = 3) and HFD-fed mice (n = 3). (D) Quantification of percentage of CGRP-positive neurons in the DRG of control (n = 3) and HFD-fed mice (n = 3). (E) Quantification of percentage of TRPV1-positive neurons in the DRG of control (n = 3) and HFD-fed mice (n = 3). Data are presented as means per mouse ± SEM. *𝑃 < 0.05, control diet vs. HFD.
(PDF)

## References

[1] Pop-Busui R, Boulton AJM, Feldman EL, Bril V, Freeman R, Malik RA, et al. Diabetic Neuropathy: A Position Statement by the American Diabetes Association. Diabetes Care. 2017;40:136–154. https://doi.org/10.2337/dc16-2042 27999003 10.2337/dc16-2042 PMC6977405

[2] Feldman EL, Nave K-A, Jensen TS, Bennett DLH. New horizons in diabetic neuropathy: mechanisms, bioenergetics, and pain. Neuron. 2017;93(6):1296–313. doi: 10.1016/j.neuron.2017.02.005 28334605 PMC5400015

[3] Themistocleous AC, Ramirez JD, Shillo PR, Lees JG, Selvarajah D, Orengo C, et al. The Pain in Neuropathy Study (PiNS): a cross-sectional observational study determining the somatosensory phenotype of painful and painless diabetic neuropathy. Pain. 2016;157(5):1132–45. doi: 10.1097/j.pain.0000000000000491 27088890 PMC4834814

[4] Margolis DJ, Malay DS, Hoffstad OJ, Leonard CE, MaCurdy T, de Nava KL, et al. Incidence of diabetic foot ulcer and lower extremity amputation among Medicare beneficiaries, 2006 to 2008: Data Points #2. Rockville (MD): Agency for Healthcare Research and Quality (US). 2011. http://www.ncbi.nlm.nih.gov/books/NBK65149/22049565

[5] Callaghan BC, Cheng HT, Stables CL, Smith AL, Feldman EL. Diabetic neuropathy: clinical manifestations and current treatments. Lancet Neurol. 2012;11(6):521–34. doi: 10.1016/S1474-4422(12)70065-0 22608666 PMC4254767

[6] Green AQ, Krishnan S, Finucane FM, Rayman G. Altered C-fiber function as an indicator of early peripheral neuropathy in individuals with impaired glucose tolerance. Diabetes Care. 2010;33(1):174–6. doi: 10.2337/dc09-0101 20040675 PMC2797968

[7] Malik RA, Tesfaye S, Newrick PG, Walker D, Rajbhandari SM, Siddique I, et al. Sural nerve pathology in diabetic patients with minimal but progressive neuropathy. Diabetologia. 2005;48(3):578–85. doi: 10.1007/s00125-004-1663-5 15729579

[8] Jolivalt CG, Frizzi KE, Guernsey L, Marquez A, Ochoa J, Rodriguez M. Peripheral neuropathy in mouse models of diabetes. Curr Protoc Mouse Biol. 2016;6:223–55. doi: 10.1002/cpmo.11 27584552 PMC5023323

[9] Sullivan KA, Hayes JM, Wiggin TD, Backus C, Su Oh S, Lentz SI, et al. Mouse models of diabetic neuropathy. Neurobiol Dis. 2007;28(3):276–85. doi: 10.1016/j.nbd.2007.07.022 17804249 PMC3730836

[10] Fontaine DA, Davis DB. Attention to background strain is essential for metabolic research: C57BL/6 and the International Knockout Mouse Consortium. Diabetes. 2016;65(1):25–33. doi: 10.2337/db15-0982 26696638 PMC4686949

[11] Heydemann A. An overview of murine high fat diet as a model for type 2 diabetes mellitus. J Diabetes Res. 2016;2016:2902351. doi: 10.1155/2016/2902351 27547764 PMC4983380

[12] Lauria G, Faber CG, Cornblath DR. Skin biopsy and small fibre neuropathies: facts and thoughts 30 years later. J Neurol Neurosurg Psychiatry. 2022;93(9):915–8. doi: 10.1136/jnnp-2021-327742 35246491 PMC9380509

[13] Abdo H, Calvo-Enrique L, Lopez JM, Song J, Zhang M-D, Usoskin D, et al. Specialized cutaneous Schwann cells initiate pain sensation. Science. 2019;365(6454):695–9. doi: 10.1126/science.aax6452 31416963

[14] Hu X, Agarwal N, Zhang M-D, Ernfors P, Kuner R, Nyengaard JR, et al. Identification and quantification of nociceptive Schwann cells in mice with and without Streptozotocin-induced diabetes. J Chem Neuroanat. 2022;123:102118. doi: 10.1016/j.jchemneu.2022.102118 35680105

[15] Ojeda-Alonso J, Calvo-Enrique L, Paricio-Montesinos R, Kumar R, Zhang MD, Poulet JFA. Sensory Schwann cells set perceptual thresholds for touch and selectively regulate mechanical nociception. 2023. doi: 10.1101/2022.02.04.477749 PMC10847425 38320986

[16] Rinwa P, Calvo-Enrique L, Zhang M-D, Nyengaard JR, Karlsson P, Ernfors P. Demise of nociceptive Schwann cells causes nerve retraction and pain hyperalgesia. Pain. 2021;162(6):1816–27. doi: 10.1097/j.pain.0000000000002169 33979318 PMC8120683

[17] Eid SA, Feldman EL. Advances in diet-induced rodent models of metabolically acquired peripheral neuropathy. Dis Model Mech. 2021;14(11):dmm049337. doi: 10.1242/dmm.049337 34762126 PMC8592018

[18] Guilford BL, Ryals JM, Wright DE. Phenotypic changes in diabetic neuropathy induced by a high-fat diet in diabetic C57BL/6 mice. Exp Diabetes Res. 2011;2011:848307. doi: 10.1155/2011/848307 22144990 PMC3226416

[19] O’Brien PD, Guo K, Eid SA, Rumora AE, Hinder LM, Hayes JM, et al. Integrated lipidomic and transcriptomic analyses identify altered nerve triglycerides in mouse models of prediabetes and type 2 diabetes. Dis Model Mech. 2020;13(2):dmm042101. doi: 10.1242/dmm.042101 31822493 PMC6994925

[20] Surwit RS, Kuhn CM, Cochrane C, McCubbin JA, Feinglos MN. Diet-induced type II diabetes in C57BL/6J mice. Diabetes. 1988;37(9):1163–7. doi: 10.2337/diab.37.9.1163 3044882

[21] Zurborg S, Piszczek A, Martínez C, Hublitz P, Al Banchaabouchi M, Moreira P, et al. Generation and characterization of an Advillin-Cre driver mouse line. Mol Pain. 2011;7:66. doi: 10.1186/1744-8069-7-66 21906401 PMC3185264

[22] Leone DP, Genoud S, Atanasoski S, Grausenburger R, Berger P, Metzger D, et al. Tamoxifen-inducible glia-specific Cre mice for somatic mutagenesis in oligodendrocytes and Schwann cells. Mol Cell Neurosci. 2003;22(4):430–40. doi: 10.1016/s1044-7431(03)00029-0 12727441

[23] Obrosova IG, Li F, Abatan OI, Forsell MA, Komjáti K, Pacher P, et al. Role of poly(ADP-ribose) polymerase activation in diabetic neuropathy. Diabetes. 2004;53(3):711–20. doi: 10.2337/diabetes.53.3.711 14988256

[24] Oh SS, Hayes JM, Sims-Robinson C, Sullivan KA, Feldman EL. The effects of anesthesia on measures of nerve conduction velocity in male C57Bl6/J mice. Neurosci Lett. 2010;483(2):127–31. doi: 10.1016/j.neulet.2010.07.076 20691755 PMC2941214

[25] Schulz A, Walther C, Morrison H, Bauer R. In vivo electrophysiological measurements on mouse sciatic nerves. J Vis Exp. 2014;(86):51181. doi: 10.3791/51181 24747166 PMC4166965

[26] Jaafar AK, Paulo-Ramos A, Rastoldo G, Veeren B, Planesse C, Bringart M, et al. PCSK9 deficiency promotes the development of peripheral neuropathy. JCI Insight. 2025;10(12):e183786. doi: 10.1172/jci.insight.183786 40338666 PMC12220952

[27] Bonin RP, Bories C, De Koninck Y. A simplified up-down method (SUDO) for measuring mechanical nociception in rodents using von Frey filaments. Mol Pain. 2014;10:26. doi: 10.1186/1744-8069-10-26 24739328 PMC4020614

[28] Hargreaves K, Dubner R, Brown F, Flores C, Joris J. A new and sensitive method for measuring thermal nociception in cutaneous hyperalgesia. Pain. 1988;32(1):77–88. doi: 10.1016/0304-3959(88)90026-7 3340425

[29] Yeomans DC, Proudfit HK. Nociceptive responses to high and low rates of noxious cutaneous heating are mediated by different nociceptors in the rat: electrophysiological evidence. Pain. 1996;68(1):141–50. doi: 10.1016/S0304-3959(96)03177-6 9252009

[30] Deuis JR, Vetter I. The thermal probe test: a novel behavioral assay to quantify thermal paw withdrawal thresholds in mice. Temperature (Austin). 2016;3(2):199–207. doi: 10.1080/23328940.2016.1157668 27857950 PMC4965000

[31] Bourane S, Grossmann KS, Britz O, Dalet A, Del Barrio MG, Stam FJ, et al. Identification of a spinal circuit for light touch and fine motor control. Cell. 2015;160(3):503–15. doi: 10.1016/j.cell.2015.01.011 25635458 PMC4431637

[32] Gerber D, Pereira JA, Gerber J, Tan G, Dimitrieva S, Yángüez E, et al. Transcriptional profiling of mouse peripheral nerves to the single-cell level to build a sciatic nerve ATlas (SNAT). eLife. 2021;10: e58591. doi: 10.7554/eLife.58591 PMC8064760 33890853

[33] Gomez-Sanchez JA, Pilch KS, van der Lans M, Fazal SV, Benito C, Wagstaff LJ, et al. After nerve injury, lineage tracing shows that myelin and remak schwann cells elongate extensively and branch to form repair schwann cells, which shorten radically on remyelination. J Neurosci. 2017;37(37):9086–99. doi: 10.1523/JNEUROSCI.1453-17.2017 28904214 PMC5597985

[34] Caterina MJ, Schumacher MA, Tominaga M, Rosen TA, Levine JD, Julius D. The capsaicin receptor: a heat-activated ion channel in the pain pathway. Nature. 1997;389(6653):816–24. doi: 10.1038/39807 9349813

[35] Vincent AM, Hayes JM, McLean LL, Vivekanandan-Giri A, Pennathur S, Feldman EL. Dyslipidemia-induced neuropathy in mice: the role of oxLDL/LOX-1. Diabetes. 2009;58(10):2376–85. doi: 10.2337/db09-0047 19592619 PMC2750230

[36] Xu L, Lin X, Guan M, Zeng Y, Liu Y. Verapamil attenuated prediabetic neuropathy in high-fat diet-fed mice through inhibiting TXNIP-mediated apoptosis and inflammation. Oxid Med Cell Longev. 2019;2019:1896041. doi: 10.1155/2019/1896041 30733849 PMC6348807

[37] Biessels GJ, Bril V, Calcutt NA, Cameron NE, Cotter MA, Dobrowsky R, et al. Phenotyping animal models of diabetic neuropathy: a consensus statement of the diabetic neuropathy study group of the EASD (Neurodiab). J Peripher Nerv Syst. 2014;19(2):77–87. doi: 10.1111/jns5.12072 24934510 PMC4303044

[38] Preguiça I, Alves A, Nunes S, Gomes P, Fernandes R, Viana SD, et al. Diet-induced rodent models of diabetic peripheral neuropathy, retinopathy and nephropathy. Nutrients. 2020;12(1):250. doi: 10.3390/nu12010250 31963709 PMC7019796

[39] Jaroslawska J, Korytko A, Zglejc-Waszak K, Antonowski T, Pomianowski AS, Wasowicz K, et al. Peripheral neuropathy presents similar symptoms and pathological changes in both high-fat diet and pharmacologically induced pre- and diabetic mouse models. Life (Basel). 2021;11(11):1267. doi: 10.3390/life11111267 34833143 PMC8618965

[40] Zaidi S, Samad AA. Assessment of nerve conduction velocity slowing and its association with the severity of diabetic polyneuropathy, duration of diabetes and glycemic control in diabetic patients. Pak J Med Sci. 2025;41(3):699–705. doi: 10.12669/pjms.41.3.10397 40103903 PMC11911748

[41] Julius D, Basbaum AI. Molecular mechanisms of nociception. Nature. 2001;413(6852):203–10. doi: 10.1038/35093019 11557989

[42] Kupari J, Ernfors P. Molecular taxonomy of nociceptors and pruriceptors. Pain. 2023;164(6):1245–57. doi: 10.1097/j.pain.0000000000002831 36718807 PMC10184562

[43] Pabbidi RM, Yu S-Q, Peng S, Khardori R, Pauza ME, Premkumar LS. Influence of TRPV1 on diabetes-induced alterations in thermal pain sensitivity. Mol Pain. 2008;4:9. doi: 10.1186/1744-8069-4-9 18312687 PMC2275252

[44] Ye D, Fairchild TJ, Vo L, Drummond PD. Pain modulation and central sensitization in painful diabetic peripheral neuropathy: updated narrative review and future directions. J Diabetes. 2026;18: e70223. doi: 10.1111/1753-0407.70223 PMC13051827 41934114

[45] Bars DL, Gozariu M, Cadden SW. Animal models of nociception. Pharmacol Rev. 2001;53:597–652. 11734620

[46] Landerholm ÅH, Hansson PT. The perception threshold counterpart to dynamic and static mechanical allodynia assessed using von Frey filaments in peripheral neuropathic pain patients. Scand J Pain. 2011;2(1):9–16. doi: 10.1016/j.sjpain.2010.08.001 29913720

[47] Hakim S, Jain A, Adamson SS, Petrova V, Indajang J, Kim HW, et al. Macrophages protect against sensory axon loss in peripheral neuropathy. Nature. 2025;640(8057):212–20. doi: 10.1038/s41586-024-08535-1 39939762 PMC11964918

[48] Eid SA, Elzinga SE, Guo K, Hinder LM, Hayes JM, Pacut CM, et al. Transcriptomic profiling of sciatic nerves and dorsal root ganglia reveals site-specific effects of prediabetic neuropathy. Transl Res. 2024;270:24–41. doi: 10.1016/j.trsl.2024.03.009 38556110 PMC11166517

[49] Mondschein AS, DiPersio MR, Zajaceskowski J, Nimmagadda H, Acheta J, Salinero AE, et al. High-fat diet disrupt nerve function by targeting schwann cells. J Peripher Nerv Syst. 2025;30(2):e70036. doi: 10.1111/jns.70036 40522084 PMC12233314

[50] Coppey L, Davidson E, Lu B, Gerard C, Yorek M. Vasopeptidase inhibitor ilepatril (AVE7688) prevents obesity- and diabetes-induced neuropathy in C57Bl/6J mice. Neuropharmacology. 2011;60(2–3):259–66. doi: 10.1016/j.neuropharm.2010.09.008 20849865 PMC3014404

[51] Coppey LJ, Holmes A, Davidson EP, Yorek MA. Partial replacement with menhaden oil improves peripheral neuropathy in high-fat-fed low-dose streptozotocin type 2 diabetic rat. J Nutr Metab. 2012;2012:950517. doi: 10.1155/2012/950517 22988492 PMC3439986

[52] Holmes A, Coppey LJ, Davidson EP, Yorek MA. Rat models of diet-induced obesity and high fat/low dose streptozotocin type 2 diabetes: effect of reversal of high fat diet compared to treatment with enalapril or menhaden oil on glucose utilization and neuropathic endpoints. J Diabetes Res. 2015;2015:307285. doi: 10.1155/2015/307285 26229968 PMC4503545

[53] Griffin TM, Fermor B, Huebner JL, Kraus VB, Rodriguiz RM, Wetsel WC, et al. Diet-induced obesity differentially regulates behavioral, biomechanical, and molecular risk factors for osteoarthritis in mice. Arthritis Res Ther. 2010;12(4):R130. doi: 10.1186/ar3068 20604941 PMC2945020

[54] Liang Y-J, Feng S-Y, Qi Y-P, Li K, Jin Z-R, Jing H-B, et al. Contribution of microglial reaction to increased nociceptive responses in high-fat-diet (HFD)-induced obesity in male mice. Brain Behav Immun. 2019;80:777–92. doi: 10.1016/j.bbi.2019.05.026 31108168

[55] Ueda H, Neyama H, Matsushita Y. Lysophosphatidic acid receptor 1- and 3-mediated hyperalgesia and hypoalgesia in diabetic neuropathic pain models in mice. Cells. 2020;9:1906. doi: 10.3390/cells9081906 32824296 PMC7465054

[56] Xu L, Tang D, Guan M, Xie C, Xue Y. Effect of high-fat diet on peripheral neuropathy in C57BL/6 mice. Int J Endocrinol. 2014;2014:305205. doi: 10.1155/2014/305205 25404943 PMC4227356

[57] Menichella DM, Abdelhak B, Ren D, Shum A, Frietag C, Miller RJ. CXCR4 chemokine receptor signaling mediates pain in diabetic neuropathy. Mol Pain. 2014;10:42. doi: 10.1186/1744-8069-10-42 24961298 PMC4078021

[58] Sajic M, Rumora AE, Kanhai AA, Dentoni G, Varatharajah S, Casey C. High dietary fat consumption impairs axonal mitochondrial function in vivo. J Neurosci. 2021;41:4321–34. doi: 10.1523/JNEUROSCI.1852-20.2021 33785643 PMC8143198

[59] George DS, Hackelberg S, Jayaraj ND, Ren D, Edassery SL, Rathwell CA, et al. Mitochondrial calcium uniporter deletion prevents painful diabetic neuropathy by restoring mitochondrial morphology and dynamics. Pain. 2022;163(3):560–78. doi: 10.1097/j.pain.0000000000002391 34232927 PMC8720329

[60] Yadlapalli JSK, Dogra N, Walbaum AW, Prather PL, Crooks PA, Dobretsov M. Pinprick hypo- and hyperalgesia in diabetic rats: can diet content affect experimental outcome?. Neurosci Lett. 2018;673:24–7. doi: 10.1016/j.neulet.2018.02.054 29490230 PMC5889730

[61] Yu Y. Gold standard for diagnosis of DPN. Front Endocrinol (Lausanne). 2021;12:719356. doi: 10.3389/fendo.2021.719356 34764937 PMC8576350

[62] Sumner CJ, Sheth S, Griffin JW, Cornblath DR, Polydefkis M. The spectrum of neuropathy in diabetes and impaired glucose tolerance. Neurology. 2003;60(1):108–11. doi: 10.1212/wnl.60.1.108 12525727

[63] Ziegler D, Papanas N, Zhivov A, Allgeier S, Winter K, Ziegler I, et al. Early detection of nerve fiber loss by corneal confocal microscopy and skin biopsy in recently diagnosed type 2 diabetes. Diabetes. 2014;63(7):2454–63. doi: 10.2337/db13-1819 24574045

[64] De Logu F, Nassini R, Materazzi S, Carvalho Gonçalves M, Nosi D, Rossi Degl’Innocenti D, et al. Schwann cell TRPA1 mediates neuroinflammation that sustains macrophage-dependent neuropathic pain in mice. Nat Commun. 2017;8(1):1887. doi: 10.1038/s41467-017-01739-2 29192190 PMC5709495

[65] Raut NGR, Maile LA, Oswalt LM, Mitxelena I, Adlakha A, Sprague KL, et al. Schwann cells modulate nociception in neurofibromatosis 1. JCI Insight. 2024;9. doi: 10.1172/jci.insight.171275 PMC10906222 38258905

