# Supplementary material for: Characterization of diabetic peripheral neuropathy in a high-fat diet mouse model
Source: PLoS One. 2026 Sep 1;21(9):e0356285. doi: 10.1371/journal.pone.0356285 (PMC13533349; doi:10.1371/journal.pone.0356285)
Supplement: Supplementary file 1 [file pone.0356285.s001.pdf]

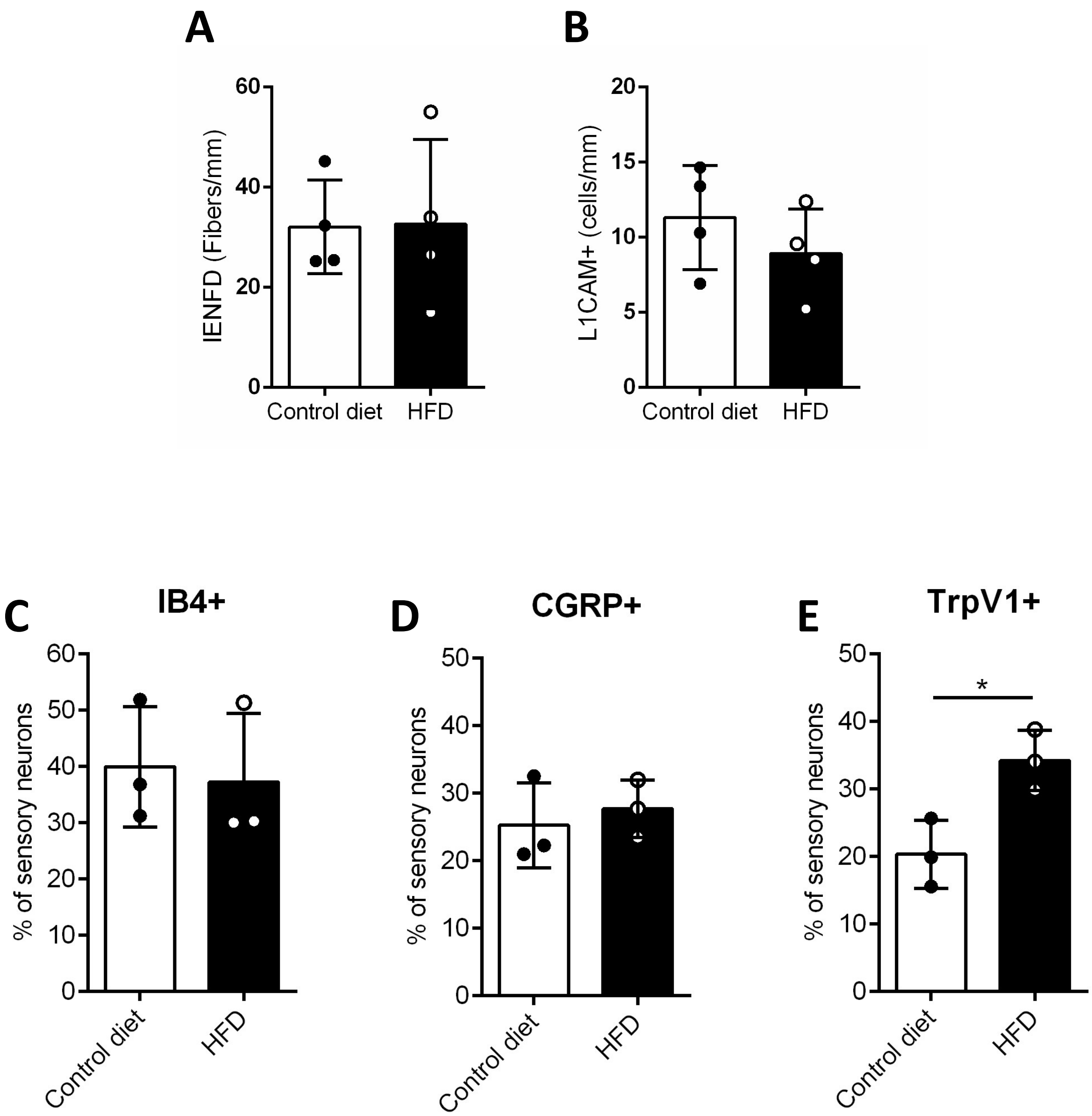

Supplementary Figure 1: Analysis of IENFD, nSCs in the foot skin and nociceptive neurons subtypes in the DRG of 12 weeks HFD-fed mice. (**A**) Quantification of IENFD in foot skin section of control (n=4) and HFD-fed mice (n=4) presented as the number of fibers/mm of epidermis. (**B**) Quantification of L1CAM-positive cells presented in number of cells/mm of epidermis in control diet (n=4) and HFD mice (n=4). (**C**) Quantification of percentage of IB4 positive neurons in the DRG of control (n=3) and HFD-fed mice (n=3). (**D**) Quantification of percentage of CGRP positive neurons in the DRG of control (n=3) and HFD-fed mice (n=3). (**E**) Quantification of percentage of TRPV1 positive neurons in the DRG of control (n=3) and HFD-fed mice (n=3). Data are presented as means per mice ± SEM. $^*P < 0.05$, control diet vs. HFD.
