# Supplementary material for: Characterization of diabetic peripheral neuropathy in a high-fat diet mouse model
Source: PLoS One. 2026 Sep 1;21(9):e0356285. doi: 10.1371/journal.pone.0356285 (PMC13533349; doi:10.1371/journal.pone.0356285)
Supplement: Peer review history

# Peer review: Decision Letter 0

**DOI:** 10.1371/journal.pone.0356285.r001

**Authors:** Alexander G Obukhov

13 Jan 2026

Dear Dr. Rastoldo,

Please submit your revised manuscript by Feb 27 2026 11:59PM. If you will need more time than this to complete your revisions, please reply to this message or contact the journal office at plosone@plos.org. When you're ready to submit your revision, log on to https://www.editorialmanager.com/pone/ and select the 'Submissions Needing Revision' folder to locate your manuscript file.

- A letter that responds to each point raised by the academic editor and reviewer(s). You should upload this letter as a separate file labeled 'Response to Reviewers'.
- A marked-up copy of your manuscript that highlights changes made to the original version. You should upload this as a separate file labeled 'Revised Manuscript with Track Changes'.
- An unmarked version of your revised paper without tracked changes. You should upload this as a separate file labeled 'Manuscript'.

If you would like to make changes to your financial disclosure, please include your updated statement in your cover letter. Guidelines for resubmitting your figure files are available below the reviewer comments at the end of this letter.

If applicable, we recommend that you deposit your laboratory protocols in protocols.io to enhance the reproducibility of your results. Protocols.io assigns your protocol its own identifier (DOI) so that it can be cited independently in the future. For instructions see: https://journals.plos.org/plosone/s/submission-guidelines#loc-laboratory-protocols. Additionally, PLOS ONE offers an option for publishing peer-reviewed Lab Protocol articles, which describe protocols hosted on protocols.io. Read more information on sharing protocols at https://plos.org/protocols?utm_medium=editorial-email&utm_source=authorletters&utm_campaign=protocols.

We look forward to receiving your revised manuscript.

Kind regards,

Alexander G Obukhov, Ph.D.

Academic Editor

PLOS One

Journal Requirements:

When submitting your revision, we need you to address these additional requirements.

1. Please ensure that your manuscript meets PLOS ONE's style requirements, including those for file naming. The PLOS ONE style templates can be found at

https://journals.plos.org/plosone/s/file?id=wjVg/PLOSOne_formatting_sample_main_body.pdf and

https://journals.plos.org/plosone/s/file?id=ba62/PLOSOne_formatting_sample_title_authors_affiliations.pdf.

2. Thank you for stating in your Funding Statement:

“This research was supported by a grant funded by Inserm, Paris, France [ATIP-AVENIR].”

Please provide an amended statement that declares *all* the funding or sources of support (whether external or internal to your organization) received during this study, as detailed online in our guide for authors at http://journals.plos.org/plosone/s/submit-now.  Please also include the statement “There was no additional external funding received for this study.” in your updated Funding Statement.

Please include your amended Funding Statement within your cover letter. We will change the online submission form on your behalf.

3. Thank you for stating the following financial disclosure:

“This research was supported by a grant funded by Inserm, Paris, France [ATIP-AVENIR].”

Please state what role the funders took in the study.  If the funders had no role, please state: "The funders had no role in study design, data collection and analysis, decision to publish, or preparation of the manuscript."

If this statement is not correct you must amend it as needed.

Please include this amended Role of Funder statement in your cover letter; we will change the online submission form on your behalf.

4. We note that your Data Availability Statement is currently as follows: [All relevant data are within the manuscript]

Please confirm at this time whether or not your submission contains all raw data required to replicate the results of your study. Authors must share the “minimal data set” for their submission. PLOS defines the minimal data set to consist of the data required to replicate all study findings reported in the article, as well as related metadata and methods (https://journals.plos.org/plosone/s/data-availability#loc-minimal-data-set-definition).

For example, authors should submit the following data:

- The values behind the means, standard deviations and other measures reported;

- The values used to build graphs;

- The points extracted from images for analysis.

Authors do not need to submit their entire data set if only a portion of the data was used in the reported study.

If your submission does not contain these data, please either upload them as Supporting Information files or deposit them to a stable, public repository and provide us with the relevant URLs, DOIs, or accession numbers. For a list of recommended repositories, please see https://journals.plos.org/plosone/s/recommended-repositories.

If there are ethical or legal restrictions on sharing a de-identified data set, please explain them in detail (e.g., data contain potentially sensitive information, data are owned by a third-party organization, etc.) and who has imposed them (e.g., an ethics committee). Please also provide contact information for a data access committee, ethics committee, or other institutional body to which data requests may be sent. If data are owned by a third party, please indicate how others may request data access.

5. If the reviewer comments include a recommendation to cite specific previously published works, please review and evaluate these publications to determine whether they are relevant and should be cited. There is no requirement to cite these works unless the editor has indicated otherwise.

[Note: HTML markup is below. Please do not edit.]

Reviewers' comments:

Reviewer's Responses to Questions

**Comments to the Author**

1. Is the manuscript technically sound, and do the data support the conclusions?

Reviewer #1: No

**********

2. Has the statistical analysis been performed appropriately and rigorously?

Reviewer #1: No

**********

3. Have the authors made all data underlying the findings in their manuscript fully available?

The PLOS Data policy

Reviewer #1: Yes

**********

4. Is the manuscript presented in an intelligible fashion and written in standard English?

Reviewer #1: Yes

**********

Reviewer #1: In the manuscript “Characterization of diabetic peripheral neuropathy in a high-fat diet mouse model,” the authors described a mouse model of diabetic peripheral neuropathy in which mice were fed a 45% high-fat diet for 16 weeks. According to their report, the mice developed obesity, hyperglycaemia, dyslipidaemia, impaired glucose tolerance, and insulin resistance. The authors also conducted tests such as von Frey, paintbrush, pinprick, and Hargreaves to assess nociceptive responses, observing that mice on the 45% high-fat diet developed mechanical hypoalgesia and thermal hyperalgesia around 12 weeks. They quantified intraepidermal nerve fibre density (IENFD) using both PGP9.5 and a transgenic mouse line, finding a significant reduction in IENF numbers. A key strength of the paper is the identification of L1CAM as a marker for nociceptive Schwann cells, which appeared decreased in high-fat diet (HFD) mice.

Overall, this study offers an interesting characterisation of the 45% HFD mouse model for diabetic peripheral neuropathy (DPN); however, several important aspects require clarification.

Major points:

1. The authors report that at 12 weeks, HFD mice developed both hypoalgesia and hyperalgesia to thermal stimuli. However, Figure 3 shows that by 16 weeks, mechanical thresholds in HFD mice return to control levels, whereas thermal hyperalgesia persists.

2. The authors should clarify whether mechanical hypoalgesia is a transient feature in this model and support this behavioural finding with histological validation (e.g., IENFD measured at different time points and Schwann cell markers).

3. This manuscript would benefit from dorsal root ganglion (DRG) characterisation, as several points remain unanswered. Are particular DRG subtypes affected? Which neuronal populations are most associated with thermal hyperalgesia in this model? Including additional sensory fiber markers could provide deeper insights.

4. It is unclear whether histological analysis was performed at 12 weeks, 16 weeks, or both. As mentioned earlier, assessing multiple time points for histology is essential for a more comprehensive understanding.

5. The authors conducted nerve conduction velocity (NCV) analysis of motor and sensory nerves, which showed no abnormalities in HFD mice. They should explain this, especially given some discrepancies with the findings in Eid et al. 2024. The authors used the same 45% HFD and measured both mechanical nociceptive responses and nerve conduction velocity at 12 weeks yet observed opposing results. While mechanical hypoalgesia was seen in this manuscript at 12 weeks, Eid et al. (2024) reported that mice initially experienced mechanical allodynia at 12 weeks, with hypoalgesia developing only at later stages. Eid et al. (2024) also reported early sural NCV slowing, which was not observed in the present study.

6. A notable aspect of this paper is the expression of L1CAM in Schwann cells of the peripheral nervous system. However, Figure 5b needs improvement, as it is difficult to determine whether the two markers truly co-localize biologically or if the pattern is merely due to spectral crosstalk.

Minor comment:

Sex should be acknowledged as a limitation. Given the increasing evidence of sex-dependent differences, the authors should acknowledge this as a potential limitation.

**********

what does this mean?). If published, this will include your full peer review and any attached files.

If you choose “no”, your identity will remain anonymous but your review may still be made public.

**Do you want your identity to be public for this peer review?**  For information about this choice, including consent withdrawal, please see our Privacy Policy

Reviewer #1: No

**********

[NOTE: If reviewer comments were submitted as an attachment file, they will be attached to this email and accessible via the submission site. Please log into your account, locate the manuscript record, and check for the action link "View Attachments". If this link does not appear, there are no attachment files.]

To ensure your figures meet our technical requirements, please review our figure guidelines: https://journals.plos.org/plosone/s/figures

You may also use PLOS’s free figure tool, NAAS, to help you prepare publication quality figures: https://journals.plos.org/plosone/s/figures#loc-tools-for-figure-preparation.

NAAS will assess whether your figures meet our technical requirements by comparing each figure against our figure specifications.

# Author comment: Author response to Decision Letter 1

**DOI:** 10.1371/journal.pone.0356285.r002

2 Jun 2026

Reviewer comments:

Reviewer #1: In the manuscript “Characterization of diabetic peripheral neuropathy in a high-fat diet mouse model,” the authors described a mouse model of diabetic peripheral neuropathy in which mice were fed a 45% high-fat diet for 16 weeks. According to their report, the mice developed obesity, hyperglycaemia, dyslipidaemia, impaired glucose tolerance, and insulin resistance. The authors also conducted tests such as von Frey, paintbrush, pinprick, and Hargreaves to assess nociceptive responses, observing that mice on the 45% high-fat diet developed mechanical hypoalgesia and thermal hyperalgesia around 12 weeks. They quantified intraepidermal nerve fibre density (IENFD) using both PGP9.5 and a transgenic mouse line, finding a significant reduction in IENF numbers. A key strength of the paper is the identification of L1CAM as a marker for nociceptive Schwann cells, which appeared decreased in high-fat diet (HFD) mice. Overall, this study offers an interesting characterisation of the 45% HFD mouse model for diabetic peripheral neuropathy (DPN); however, several important aspects require clarification.

Major points:

1. The authors report that at 12 weeks, HFD mice developed both hypoalgesia and hyperalgesia to thermal stimuli. However, Figure 3 shows that by 16 weeks, mechanical thresholds in HFD mice return to control levels, whereas thermal hyperalgesia persists.

2. The authors should clarify whether mechanical hypoalgesia is a transient feature in this model and support this behavioural finding with histological validation (e.g., IENFD measured at different time points and Schwann cell markers).

As perfectly noted by the reviewer, our data indicates that HFD-fed mice exhibit mechanical hypoalgesia at week 12 but not at week 16.

This observation is not sufficient to demonstrate either recovery or a transient phenotype, it rather illustrates the inherent variability of mechanical sensory outcomes in mouse models of HFD-induced neuropathy, similarly to other studies (Eid et al. (2024), Hakim et al. (2025), Mondschein et al. (2025)).

To clarify whether mechanical hypoalgesia is transient (or not) we have performed the measurement of IENFD and nSCs number at week 12 requested by the reviewer (See our new supplemental Figure 1A-B and results paragraph 4). We found a significant difference at week 16 in terms of IENFD and nSCs number between HFD and chow fed mice, but not at week 12.

We now discuss our results as follows (From line 397): << In addition, we observed a transient decrease of responses to mechanical stimulation induced by von-Frey filament indentations at 12 weeks but not 16 weeks post HFD.... In contrast, we did not observe any significant decrease in IENFD or nSCs number in the foot skin that could explain the decrease in mechanical sensitivity in mice fed with HFD for 12 weeks. It is noteworthy that transient mechanical alterations have also been observed in different mouse models of diabetic neuropathy [44,45] but the cellular and molecular basis of these changes are still undetermined. Future studies targeting mechanosensory neurons are necessary to better understand the mechanisms underlying this variation of mechanical sensitivity during early diabetes. >>

Eid SA, Elzinga SE, Guo K, Hinder LM, Hayes JM, Pacut CM, et al. Transcriptomic profiling of sciatic nerves and dorsal root ganglia reveals site-specific effects of prediabetic neuropathy. Transl Res J Lab Clin Med. 2024;270: 24–41. doi:10.1016/j.trsl.2024.03.009

Hakim S, Jain A, Adamson SS, Petrova V, Indajang J, Kim HW, et al. Macrophages protect against sensory axon loss in peripheral neuropathy. Nature. 2025;640: 212–220. doi:10.1038/s41586-024-08535-1

Mondschein AS, DiPersio MR, Zajaceskowski J, Nimmagadda H, Acheta J, Salinero AE, Haslam S, Poitelon E, Elston S, McFarland E, Beck B, Zuloaga KL, Rumora AE, Poitelon Y, Belin S. High-Fat Diet Disrupt Nerve Function by Targeting Schwann Cells. J Peripher Nerv Syst. 2025 Jun;30(2):e70036. doi: 10.1111/jns.70036.

3. This manuscript would benefit from dorsal root ganglion (DRG) characterization, as several points remain unanswered. Are particular DRG subtypes affected? Which neuronal populations are most associated with thermal hyperalgesia in this model? Including additional sensory fiber markers could provide deeper insights.

We thank the reviewer for this valuable suggestion and agree that further characterization of sensory neuron subtypes can provide important mechanistic insights. Following this recommendation, we performed additional analyses targeting specific nociceptor populations at 12 and 16 weeks HFD-fed mice. In dorsal root ganglia (DRG), we examined classical markers of nociceptive subtypes, including CGRP to label peptidergic nociceptors and IB4 to identify non-peptidergic nociceptors. Given that the most robust and persistent behavioral phenotype observed in our model was thermal hyperalgesia, we chose to analyze TRPV1 expression as a marker of thermosensitive nociceptors. These analyses are presented in Figure 6 (16 weeks post HFD) and supplementary Figure 1 (C-E) (12 weeks post HFD) in the revised version of the manuscript.

We did not observe any significant change in the number of non-peptidergic (Figure 6 A-C, Supplementary Figure 1C,) and peptidergic (Figure 6 D-F, Supplementary Figure 1D) nociceptive neurons after 12 and 16 weeks of HFD. We observed a significant increase in the number of neurons expressing TRPV1, at 12 weeks (Supplementary Figure 1E) but not at 16 weeks post HFD (Figure 6 G-I). Altogether, these observations indicate that nociceptive neurons subtypes in the DRG are preserved after 12 and 16 weeks of HFD with a transient increase in TRPV1 expression only after 12 weeks of diet.

Taking into consideration the results obtained, we have revised the Results section (from line 348 to 360) and Discussion section (Line 389 to 396)

4. It is unclear whether histological analysis was performed at 12 weeks, 16 weeks, or both. As mentioned earlier, assessing multiple time points for histology is essential for a more comprehensive understanding.

All histological analyses were initially performed at a single, well-established neuropathic time point, namely after 16 weeks of ND or HFD feeding. We have now systematically performed the analyses also at week 12. This is now clearly indicated in the methods and results section as well as in the revised figures.

5. The authors conducted nerve conduction velocity (NCV) analysis of motor and sensory nerves, which showed no abnormalities in HFD mice. They should explain this, especially given some discrepancies with the findings in Eid et al. 2024. The authors used the same 45% HFD and measured both mechanical nociceptive responses and nerve conduction velocity at 12 weeks yet observed opposing results. While mechanical hypoalgesia was seen in this manuscript at 12 weeks, Eid et al. (2024) reported that mice initially experienced mechanical allodynia at 12 weeks, with hypoalgesia developing only at later stages. Eid et al. (2024) also reported early sural NCV slowing, which was not observed in the present study.

We thank the reviewer for this important comment and for highlighting the comparison with Eid et al. (2024). Although both studies employed a 45% high-fat diet, the diets are not totally identical. In the present study, mice were fed a 45% fat diet from Specialty Feeds, whereas Eid et al. used a different commercially available formulation (D12451, 45% fat kcal, Research Diets). As discussed in the manuscript, even small variations in diet composition, fat source, or micronutrient content can significantly influence metabolic and neuropathic outcomes. Our observations are in line with previous work demonstrating that pain-related phenotypes in experimental diabetes models are highly sensitive to diet composition and environmental factors. Notably, Yadlapalli et al. (2018) reported a phenotypic switch from hypoalgesia to hyperalgesia in diabetic rodents that coincided with a change in diet formulation at the same vendor facility, underscoring the profound impact of diet on neuropathy progression.

In addition, the timing of HFD exposure differs substantially between the two studies. In our work, mice were placed on ND or HFD at 8 weeks of age, when the peripheral and central nervous system is fully mature, and were analyzed after 4, 8, 12 and 16 weeks of diet. In contrast, Eid et al. initiated HFD feeding at 3 weeks of age, a critical developmental period for sensory neurons and myelination (Barrera et al., 2013, Chandran et al., 1994), and performed phenotypic assessments at 12 and 36 weeks of age ie after 9 and 33 weeks of HFD. Early-life exposure to metabolic stress may therefore lead to distinct sensory dysfunctions and nerve conduction deficits compared to adult-onset HFD. These methodological differences are likely to contribute to the divergent behavioral phenotypes reported at comparable time points.

Regarding nerve conduction velocity, the absence of significant NCV slowing in our study is consistent with several reports showing that electrophysiological deficits often emerge later than behavioral and small-fiber alterations in diet-induced models of neuropathy. This dissociation between functional sensory changes and large-fiber electrophysiological measures further supports the idea that NCV may be relatively preserved during early stages of HFD-induced neuropathy.

Yadlapalli JSK, Dogra N, Walbaum AW, Prather PL, Crooks PA, Dobretsov M. Pinprick hypo- and hyperalgesia in diabetic rats: Can diet content affect experimental outcome? Neurosci Lett. 2018;673: 24–27. doi:10.1016/j.neulet.2018.02.054

Barrera K, Chu P, Abramowitz J, Steger R, Ramos RL, Brumberg JC. Organization of Myelin in the Mouse Somatosensory Barrel Cortex and the Effects of Sensory Deprivation. Dev Neurobiol. 2013;73: 297–314. doi:10.1002/dneu.22060

Chandran AP, Oda K, Shibasaki H, Pisharodi M. Spinal somatosensory evoked potentials in mice and their developmental changes. Brain Dev. 1994;16: 44–51. doi:10.1016/0387-7604(94)90112-0

6. A notable aspect of this paper is the expression of L1CAM in Schwann cells of the peripheral nervous system. However, Figure 5b needs improvement, as it is difficult to determine whether the two markers truly co-localize biologically or if the pattern is merely due to spectral crosstalk.

We thank the reviewer for raising this important point regarding the interpretation of L1CAM expression and potential spectral crosstalk in Figure 5b.

L1CAM is known to be expressed in axons and Schwann cells during embryonic and early postnatal development and remains expressed in non-myelinated Schwann in the adult (PMID: 6467033, PMID: 10477768, PMID: 2420673, PMID: 2453520). Nociceptive Schwann cells are a non-myelinated Schwann cells subtypes that protect and metabolically support nociceptive axons and can be visualized using PLP-CreERT2 mice (Abdo et al 2019). As a marker for non-myelinating Schwann cells, it’s not so surprising to find co-expression of L1CAM with PLP-CreERT2;R26TdTomato at the level of nociceptive Schwann cells.

In this study, we used a confocal microscope Eclipse Ti2 (Nikon) for imaging. In Figure 5b, we used Alexa 488 (green) and Alexa 594 (red) as fluorophores to detect L1CAM and tdTomato respectively. These two fluorophores have few overlapping emission spectra and we used the sequential acquisition mode on the confocal to avoid spectral crosstalk.

Based on these elements, we are confident that the co-expression of L1CAM with tdTomato is biological and not due to spectral crosstalk. Figure 5b has been updated accordingly in the revised version of the manuscript.

Minor comment:

Sex should be acknowledged as a limitation. Given the increasing evidence of sex-dependent differences, the authors should acknowledge this as a potential limitation.

We have added the following sentence at the end of the conclusion in accordance with the reviewer’s comment: “One limitation of this study is that all experiments were performed in male mice, and potential sex-dependent differences in the development of HFD-induced peripheral neuropathy were not addressed.

Attachment Submitted filename: Response reviewers PlosOne.docx

# Peer review: Decision Letter 1

**DOI:** 10.1371/journal.pone.0356285.r003

**Authors:** Alexander G Obukhov

2 Aug 2026

Characterization of diabetic peripheral neuropathy in a high-fat diet mouse model

PONE-D-25-60376R1

Dear Dr. Rastoldo,

We’re pleased to inform you that your manuscript has been judged scientifically suitable for publication and will be formally accepted for publication once it meets all outstanding technical requirements.

Within one week, you’ll receive an e-mail detailing the required amendments. When these have been addressed, you’ll receive a formal acceptance letter and your manuscript will be scheduled for publication.

An invoice will be generated when your article is formally accepted. Please note, if your institution has a publishing partnership with PLOS and your article meets the relevant criteria, all or part of your publication costs will be covered. Please make sure your user information is up-to-date by logging into Editorial Manager at Editorial Manager® and clicking the ‘Update My Information' link at the top of the page. For questions related to billing, please contact billing support.

If your institution or institutions have a press office, please notify them about your upcoming paper to help maximize its impact. If they’ll be preparing press materials, please inform our press team as soon as possible -- no later than 48 hours after receiving the formal acceptance. Your manuscript will remain under strict press embargo until 2 pm Eastern Time on the date of publication. For more information, please contact onepress@plos.org.

Kind regards,

Alexander G Obukhov, Ph.D.

Academic Editor

PLOS One

Reviewers' comments:

Reviewer's Responses to Questions

**Comments to the Author**

Reviewer #1: All comments have been addressed

**********

2. Is the manuscript technically sound, and do the data support the conclusions?

Reviewer #1: Yes

**********

3. Has the statistical analysis been performed appropriately and rigorously?

Reviewer #1: Yes

**********

4. Have the authors made all data underlying the findings in their manuscript fully available?

The PLOS Data policy

Reviewer #1: Yes

**********

5. Is the manuscript presented in an intelligible fashion and written in standard English?

Reviewer #1: Yes

**********

Reviewer #1: The authors have satisfactorily addressed all of my concerns. The manuscript has improved substantially and, in its current form, meets the standards for publication in PLOS ONE.

**********

what does this mean?). If published, this will include your full peer review and any attached files.

If you choose “no”, your identity will remain anonymous but your review may still be made public.

**Do you want your identity to be public for this peer review?**  For information about this choice, including consent withdrawal, please see our Privacy Policy

Reviewer #1: No

**********

# Editor report: Acceptance letter

**DOI:** 10.1371/journal.pone.0356285.r004

**Authors:** Alexander G Obukhov

PONE-D-25-60376R1

PLOS One

Dear Dr. Rastoldo,

I'm pleased to inform you that your manuscript has been deemed suitable for publication in PLOS One. Congratulations! Your manuscript is now being handed over to our production team.

At this stage, our production department will prepare your paper for publication. This includes ensuring the following:

* All references, tables, and figures are properly cited

* All relevant supporting information is included in the manuscript submission,

* There are no issues that prevent the paper from being properly typeset

You will receive further instructions from the production team, including instructions on how to review your proof when it is ready. Please keep in mind that we are working through a large volume of accepted articles, so please give us a few days to review your paper and let you know the next and final steps.

Lastly, if your institution or institutions have a press office, please let them know about your upcoming paper now to help maximize its impact. If they'll be preparing press materials, please inform our press team within the next 48 hours. Your manuscript will remain under strict press embargo until 2 pm Eastern Time on the date of publication. For more information, please contact onepress@plos.org.

You will receive an invoice from PLOS for your publication fee after your manuscript has reached the completed accept phase. If you receive an email requesting payment before acceptance or for any other service, this may be a phishing scheme. Learn how to identify phishing emails and protect your accounts at https://explore.plos.org/phishing.

If we can help with anything else, please email us at customercare@plos.org.

Thank you for submitting your work to PLOS One and supporting open access.

Kind regards,

PLOS ONE Editorial Office Staff

on behalf of

Dr. Alexander G Obukhov

Academic Editor

PLOS One
